# Supplementary material for: Random rotational embedding Bayesian optimization for human-in-the-loop personalized music generation
Source: PLoS One. 2025 Nov 21;20(11):e0335853. doi: 10.1371/journal.pone.0335853 (PMC12637961; doi:10.1371/journal.pone.0335853)
Supplement: Attachment — Submitted filename: 0160_001.pdf [file pone.0335853.s001.pdf]

PLOS ONE

Random rotational embedding Bayesian optimization for human-in-the-loop personalized music generation

--Manuscript Draft--

**Manuscript Number:**	PONE-D-25-27090
**Article Type:**	Research Article
**Full Title:**	Random rotational embedding Bayesian optimization for human-in-the-loop personalized music generation
**Short Title:**	Random rotational embedding Bayesian optimization for human-in-the-loop personalized music generation
**Corresponding Author:**	Miguel Marcos<br>UNIZAR: Universidad de Zaragoza<br>Zaragoza, SPAIN
**Keywords:**	Bayesian optimization; high-dimensional optimization; music generation; human-in-the-loop optimization
**Abstract:**	Music generation is a highly subjective task. Finding a good piece, for some given listener and situation, is challenging, but for a given piece, it is easy for the listener to rate it. Thus, the problem of generating personalized music can be modeled as an optimization problem based on listener ratings. In this work, we find a point in the latent space of a music generation model that produces a song that maximizes a human-given evaluation, which makes for a high-dimensional and heterogeneous scenario. Furthermore, human ratings are limited and expensive to gather, thus, data efficiency is fundamental. State-of-the-art solutions for feedback-based global optimization usually leverage Bayesian optimization, due to its data efficiency, and assume box-bounded or linear constraints. However, generative models often work over Gaussian latent spaces, causing box-bounded optimization to underperform. Our proposal for high-dimensional Bayesian optimization works by embedding a low-dimensional Gaussian search space into a high-dimensional one through random rotations. We evaluate our generation and optimization pipeline using synthetic data, evaluated on musical feature metrics, where we show how it outperforms other high-dimensional Bayesian optimization alternatives. Finally, we conduct an experiment on sixteen volunteers to find a generated piece of music personalized for each subject. For that, we ask them to rate a series of songs based on their taste and our pipeline generates the most informative song to rate next in order to reduce the number of queries to the user. Half of the volunteers used our optimization pipeline while half of them used songs generated with a random seed as a control group. Our results during the optimization and a posterior survey show greater performance of the optimization pipeline and a high degree of satisfaction, even for users with a very specific taste.
**Order of Authors:**	Miguel Marcos<br>Lorenzo Mur-Labadia<br>Ruben Martinez-Cantin
**Additional Information:**	
Question	Response
**Financial Disclosure**<br><br>Enter a financial disclosure statement that describes the sources of funding for the work included in this submission. Review the submission guidelines for detailed requirements. View published research articles from *PLOS ONE* for specific examples.	Yes

Powered by Editorial Manager® and ProduXion Manager® from Aries Systems Corporation

This statement is required for submission and **will appear in the published article** if the submission is accepted. Please make sure it is accurate.

Funded studies

Enter a statement with the following details:

- Initials of the authors who received each award
- Grant numbers awarded to each author
- The full name of each funder
- URL of each funder website
- Did the sponsors or funders play any role in the study design, data collection and analysis, decision to publish, or preparation of the manuscript?

Did you receive funding for this work?

Please add funding details. as follow-up to "**Financial Disclosure**	This research was funded by Spanish government projects PID2021-125209OB-I00 (https://www.aei.gob.es/) and the Aragon Government DGA T45_23R (https://www.aragon.es/).

Enter a financial disclosure statement that describes the sources of funding for the work included in this submission. Review the submission guidelines for detailed requirements. View published research articles from *PLOS ONE* for specific examples.

This statement is required for submission and **will appear in the published article** if the submission is accepted. Please make sure it is accurate.

Funded studies

Enter a statement with the following details:

- Initials of the authors who received each award
- Grant numbers awarded to each author
- The full name of each funder
- URL of each funder website
- Did the sponsors or funders play any role in the study design, data collection and analysis, decision to publish, or preparation of the manuscript?

Powered by Editorial Manager® and ProduXion Manager® from Aries Systems Corporation

Did you receive funding for this work?"	
Please select the country of your main research funder (please select carefully as in some cases this is used in fee calculation).<br>as follow-up to "**Financial Disclosure**<br><br>Enter a financial disclosure statement that describes the sources of funding for the work included in this submission. Review the submission guidelines for detailed requirements. View published research articles from *PLOS ONE* for specific examples.<br><br>This statement is required for submission and **will appear in the published article** if the submission is accepted. Please make sure it is accurate.<br><br>**Funded studies**<br>Enter a statement with the following details:<br>• Initials of the authors who received each award<br>• Grant numbers awarded to each author<br>• The full name of each funder<br>• URL of each funder website<br>• Did the sponsors or funders play any role in the study design, data collection and analysis, decision to publish, or preparation of the manuscript?<br><br>Did you receive funding for this work?"	SPAIN - ES
**Competing Interests**<br><br>Use the instructions below to enter a competing interest statement for this submission. On behalf of all authors, disclose any competing interests that could be perceived to bias this work—acknowledging all financial support and any other relevant financial or non-financial competing interests.<br><br>This statement is required for submission	The authors have declared that no competing interests exist.

and **will appear in the published article** if the submission is accepted. Please make sure it is accurate and that any funding sources listed in your Funding Information later in the submission form are also declared in your Financial Disclosure statement.

View published research articles from *PLOS ONE* for specific examples.

NO authors have competing interests

Enter: *The authors have declared that no competing interests exist.*

Authors with competing interests

Enter competing interest details beginning with this statement:

I have read the journal's policy and the authors of this manuscript have the following competing interests: [insert competing interests here]

* typeset

**Ethics Statement**<br><br>Enter an ethics statement for this submission. This statement is required if the study involved:<br><br>• Human participants<br>• Human specimens or tissue<br>• Vertebrate animals or cephalopods<br>• Vertebrate embryos or tissues<br>• Field research<br><br>Write "N/A" if the submission does not require an ethics statement.<br><br>General guidance is provided below. Consult the submission guidelines for detailed instructions. **Make sure that all information entered here is included in the Methods section of the manuscript.**	The research process was conducted according to the ethical recommendations of the Declaration of Helsinki and was approved by both our institution's data protection unit and the Aragón Autonomous Community Research Ethics Committee (CEICA), which supervises fairness in research projects and the use of biological and personal data. Our protocol is non-invasive and involves the gathering of opinion data only, with no risk for any participant, who gave their informed written consent right after explaining the procedure. The consent allowed the abandonment of the experiment, and all data was analyzed anonymously.

Powered by Editorial Manager® and ProduXion Manager® from Aries Systems Corporation

Format for specific study types

Human Subject Research (involving human participants and/or tissue)

- Give the name of the institutional review board or ethics committee that approved the study
- Include the approval number and/or a statement indicating approval of this research
- Indicate the form of consent obtained (written/oral) or the reason that consent was not obtained (e.g. the data were analyzed anonymously)

Animal Research (involving vertebrate animals, embryos or tissues)

- Provide the name of the Institutional Animal Care and Use Committee (IACUC) or other relevant ethics board that reviewed the study protocol, and indicate whether they approved this research or granted a formal waiver of ethical approval
- Include an approval number if one was obtained
- If the study involved *non-human primates*, add *additional details* about animal welfare and steps taken to ameliorate suffering
- If anesthesia, euthanasia, or any kind of animal sacrifice is part of the study, include briefly which substances and/or methods were applied

Field Research

Include the following details if this study involves the collection of plant, animal, or other materials from a natural setting:

- Field permit number
- Name of the institution or relevant body that granted permission

**Data Availability**<br><br>Authors are required to make all data underlying the findings described fully available, without restriction, and from the time of publication. PLOS allows rare exceptions to address legal and ethical concerns. See the PLOS Data Policy and FAQ for detailed information.	Yes - all data are fully available without restriction

A Data Availability Statement describing where the data can be found is required at submission. Your answers to this question constitute the Data Availability Statement and **will be published in the article**, if accepted.<br><br>**Important:** Stating 'data available on request from the author' is not sufficient. If your data are only available upon request, select 'No' for the first question and explain your exceptional situation in the text box.<br><br>Do the authors confirm that all data underlying the findings described in their manuscript are fully available without restriction?	
**Describe where the data may be found in full sentences. If you are copying our sample text, replace any instances of XXX with the appropriate details.**<br><br>• If the data are **held or will be held in a public repository**, include URLs, accession numbers or DOIs. If this information will only be available after acceptance, indicate this by ticking the box below. For example: *All XXX files are available from the XXX database (accession number(s) XXX, XXX.)*.<br>• If the data are all contained **within the manuscript and/or Supporting Information files**, enter the following: *All relevant data are within the manuscript and its Supporting Information files.*<br>• If neither of these applies but you are able to provide **details of access elsewhere**, with or without limitations, please do so. For example:<br><br>*Data cannot be shared publicly because of [XXX]. Data are available from the XXX Institutional Data Access / Ethics Committee (contact via XXX) for researchers who meet the criteria for access to confidential data.*<br><br>*The data underlying the results presented in the study are available from (include the name of the third party*	All data and code developed for this project, evaluation of the method and recreation of results will be released on a GitHub public repository under the URL 'https://github.com/mikceroese/ROMBO' after acceptance.

and contact information or URL).

- This text is appropriate if the data are owned by a third party and authors do not have permission to share the data.

* typeset

Additional data availability information:	Tick here if the URLs/accession numbers/DOIs will be available only after acceptance of the manuscript for publication so that we can ensure their inclusion before publication.

Powered by Editorial Manager® and ProduXion Manager® from Aries Systems Corporation

Random rotational embedding Bayesian optimization for human-in-the-loop personalized music generation

Miguel Marcos*, Lorenzo Mur-Labadia, Ruben Martinez-Cantin

Departamento de Informática e Ingeniería de Sistemas,
Instituto de Investigación en Ingeniería de Aragón (I3A),
Universidad de Zaragoza, Zaragoza, Spain

*m.marcos@unizar.es

Abstract

Music generation is a highly subjective task. Finding a good piece, for some given listener and situation, is challenging, but for a given piece, it is easy for the listener to rate it. Thus, the problem of generating personalized music can be modeled as an optimization problem based on listener ratings. In this work, we find a point in the latent space of a music generation model that produces a song that maximizes a human-given evaluation, which makes for a high-dimensional and heterogeneous scenario. Furthermore, human ratings are limited and expensive to gather, thus, data efficiency is fundamental. State-of-the-art solutions for feedback-based global optimization usually leverage Bayesian optimization, due to its data efficiency, and assume box-bounded or linear constraints. However, generative models often work over Gaussian latent spaces, causing box-bounded optimization to underperform. Our proposal for high-dimensional Bayesian optimization works by embedding a low-dimensional Gaussian search space into a high-dimensional one through random rotations. We evaluate our generation and optimization pipeline using synthetic data, evaluated on musical feature metrics, where we show how it outperforms other high-dimensional Bayesian optimization alternatives. Finally, we conduct an experiment on sixteen volunteers to find a generated piece of music personalized for each subject. For that, we ask them to rate a series of songs based on their taste and our pipeline generates the most informative song to rate next in order to reduce the number of queries to the user. Half of the volunteers used our optimization pipeline while half of them used songs generated with a random seed as a control group. Our results during the optimization and a posterior survey show greater performance of the optimization pipeline and a high degree of satisfaction, even for users with a very specific taste.

Introduction

Music generation systems can be useful tools in a wide range of applications, such as initiating amateur musicians [1–5], helping in music therapy for medical treatments [6, 7], and even remixing or rearranging songs at a user's whim. However, most approaches fail to take into account individual taste, like some prompt-based models [1, 2] that can follow general guidelines but require heavy prompt engineering to achieve the desired rhythm, tension, or emotion flow [8]. Nonetheless, taste is difficult to generalize, as music is highly subjective. Therefore, a music generation pipeline should have two key components: first, a generative model that can provide a broad and

potentially complex enough variety of results. Second, a tuning phase that shifts these results to the desired features using human feedback. This is similar to how modern large-language models (LLMs) complement their pre-trained architectures with reinforcement learning with human feedback (RLHF) [9, 10]. In these models, the large generative model is fine-tuned to optimize the reward estimated from previous interactions with humans to address their common preferences. Meanwhile, our approach is intended to personalize results when the optimization or fine-tuning must be done with human-in-the-loop data from a single individual, focusing on data efficiency.

Bayesian optimization (BO) is the state-of-the-art approach for data-efficient black-box global optimization [11–13] and, therefore, it excels in human-feedback in-the-loop optimization [14–18]. BO combines a surrogate model built iteratively on observations and an acquisition function that locates potential optima, focusing the search around promising candidates. The surrogate model acts as a memory of previous queries, quickly reducing the search space as we gather data. Meanwhile, the acquisition function uses the surrogate model's information combined with optimal decision theory, which results in a trade-off between exploration and exploitation of the search space. BO is also robust to observation noise, which is a typical limitation of human feedback. By using a probabilistic surrogate model, we can encode and take into account noise in the query responses and provide a robust solution, even in the presence of spurious data [19] or mismodeling errors [20].

A common issue of global optimization, including BO, is the curse of dimensionality. High-dimensional problems require an exponentially large number of observations or a strong prior-over-the-problem structure [11]. In practice, most strategies are typically limited to low-dimensional problems and fail to address problems with hundreds of dimensions in the search space. Under certain conditions, we can apply BO for high dimensional problems if we assume that they have structural properties that we can exploit, such that, the effective dimensionality of the problem is within a low dimensional manifold [21, 22], the problem can be decomposed in additive subspaces [23, 24] or we can address the problem with local models [25]. In this paper, we present a comparison of different approaches focused on the specific problem of harmonic music generation featuring several instruments, a most complex scenario. Furthermore, we propose a novel approach that tackles the specific challenge of high-dimensional Gaussian search spaces of generative models for Bayesian optimization.

We build a pipeline that maps a user's numerical evaluation to the different possible generations of a model and their latent space representation. By reducing the dimensionality of the model's latent space and wrapping it together with the generative process and human evaluation, we can assemble a target function to optimize through BO and find a generation among the model's range that best caters to an unknown user's taste.

To prove the effectiveness of the pipeline, we design and perform a series of human trials. We gather a total of sixteen participants who are tasked with grading a gold-standard music generation model's outputs, intending to find one that maximizes its evaluation within a set limit of trials. Comparing the features of the users' preferred generations and their opinions, which we gather through a Likert scale survey, we find evidence of the need for optimization and of the method's performance. This work is, to our knowledge, the second to apply BO to music generation with human feedback [26], and the first one to ever try to optimize high-complexity harmonic compositions for several instruments.

Our main contributions are:

- A sample-efficient, personalized music generation method based on subjective evaluations.
- A novel approach for performing Bayesian optimization in high-dimensional

Fig 1. Overview of our proposed pipeline. We sample a low-dimensional search space, which is remapped onto a music generation model's high-dimensional latent space. After playing the resultant piece, we use a user-given grade to build a surrogate model and efficiently search for new candidate points using Bayesian Optimization, adapting to a user's taste in real time without retraining the model.

Gaussian search spaces, especially useful for generative models, outperforming other Bayesian optimization baselines and dimensionality reduction techniques.

- The implementation of a pipeline using Bayesian optimization and our own version of a gold-standard music generation model.
- A collection of human-driven experiments proving both the need for model personalization and the effectiveness of our method.

Related works

Generative models

The goal of a generative model is to capture the distribution of a set of data and then be able to create samples within said distribution. Within this category, we find Generative Adversarial Networks [27] (GANs): their later successors, Wasserstein GANs [28] (WGANs); and Variational Autoencoders [29, 30] (VAEs). All these architectures use two networks that either compete or collaborate with each other and ultimately create a latent space to sample from later. The last two of them are employed in our method. Another kind, diffusion models [31], learn to generate data from noise by learning a denoising function by inverting the process of adding noise, conditioning the process on a target class, descriptor or prompt.

Though these models are widely used for image generation, other data types, like text or music, can be generated with them, and there are specialized architectures for different data types. For example, Generative Pretrained Transformers (GPTs [32, 33]) exploit attention mechanisms [34] to generate text word by word, conditioning it on both a prompt and their previously generated text. They are often tuned with Reinforcement Learning from Human Feedback [9, 10, 35] (RLHF), which profiles preferred outputs based on human judgment, in a way similar to our proposed method.

For the particular case of artificial music composition and generation, the first artificial neural networks used for composition started with MultiLayered Perceptrons (MLPs) [36, 37], then evolved to Short-Term Memory Networks [38, 39] (LSTMs) and into GANs [40, 41]. Found among the latter, MuseGAN [40] is a WGAN trained over MIDI files to create piano rolls, binary matrices with positive values representing each sounding note, with up to five different instruments. Coconet [42], a later model that also works on composition, approaches the process by trying to imitate human composers. Instead of writing in the direction of time like other models, a Neural Autoregressive Density Estimator (NADE) [43] fills gaps in Bach chorales. This teaches the model to rewrite and iterate over its pieces, not tying the process to any causal direction. Later models shift focus from the symbolic domain to the audio domain, abstracting the composition process. MelNet [44], a model contemporary to Coconet, generates realistic piano solos and is trained with Mel-spectrograms, 2D decompositions of the soundwave on different frequencies remapped to a quasi-logarithmical perception-based scale. Later in time, we encounter models that exploit the Transformer architecture, like AudioCraft, which combines audio compression [3] and text-to-sound generation [4] [5], or Jukebox [1], which can also generate vocal parts from

written lyrics. Lastly, one of the latest trending examples is Bark [2], which is the text-to-speech model used in the AI music generator Suno and is capable of generating music and sound effects next to the lyrics from the text prompt.

Bayesian optimization

Bayesian optimization has been widely used in many applications and scenarios. Examples include hyperparameter tuning for neural networks [45], robust policy search, planning, and motion in robotics [20, 46, 47], autonomous systems [12], game agent optimization [48], aircraft design [49], physics modeling and simulation [50], biochemistry [51], ecology [52], and medicine [53, 54] among others.

Some works focus on Bayesian optimization as a way to take into account human judgment for tasks like image generation [55], photography color enhancement [16, 56], material appearance modeling [16, 57], procedural animations [17] and prosthesis calibration [15, 58]. These cases usually lean towards preferential Bayesian optimization (PBO), which captures relations between evaluations instead of assigning scalars individually. This takes care of some biases, such as drift or anchoring, when working with human judgment. PBO has been used for the optimization of music generation [26], where users manipulate an interface for melody composition, onto which they can select a two-bar section for the system to propose variations, which they can also edit themselves. However, high-dimensional PBO is still an open problem with few preliminary results addressing it [59] limited to 1D preferences.

High-dimensional vanilla BO, on the other hand, is a well-known issue, and many techniques tackle its limitations [60]. Some assume the effective dimensionality of the problem is smaller than its domain and hence some dimensions, which may be axis aligned [61, 62] or not [21, 22], may be uninformative, so they opt to cull down the search space. Another option is breaking down the GP into a sum of several smaller and independent component functions, which receives the name of Additive kernels [24, 63, 64]. This approach makes the total complexity of fitting all kernels lower than fitting a single global kernel. Some others choose to change the exploration strategy. Some works propose to impose locality on the optimization [25], which simplifies the process, while others prefer transforming the geometry of the search space by making it non-Euclidean, e.g., through cylindrical kernels [65], which effectively expands the center of the search space while shrinking the borders.

Methods

We present a pipeline for personalized music generation leveraging human feedback. Our approach, portrayed in Figure 1, has four essential pieces: A deep-learning model for music generation, Bayesian optimization as our sample-efficient query generation for finding a high-quality personalized composition, a dimensionality reduction process to make the optimization tractable, and a user interface to evaluate the queries (compositions) based on user taste. Below, we explain the details of each of these main parts.

Music generation model

Our method's foundation is a music generation model. Following former literature [26] on music-oriented BO, we chose symbolic music generation as the domain of our problem.

Symbolic music is most often represented in score sheets: Several instruments play different parts of the score, each with their own dynamics, but interacting with each

other. Notes unfold along time following structural patterns, grouping in bars and forming phrases. Lastly, notes are played in different pitches, and stack forming arpeggios and chords.

Our data is a set of piano rolls, which imitate the structure of score sheets. A piano roll is a multi-dimensional binary tensor, which is made of I instrument tracks that play for B bars of T timesteps. Instruments play within a range of P pitches, with zeros meaning silence, and ones meaning sound. Thus, a piano roll is defined as $\Psi \in [0,1]^{I \times B \times T \times P}$. This multi-dimensional setup allows for several instruments to play several notes at the same time. Compared to melodies, where only up to one note is playing at a time, it increases the range of possible generations, as well as complexity.

For our selected model, we take inspiration from MuseGAN [40], a WGAN capable of generating piano rolls for up to five instruments. Formally, a WGAN is composed of two networks, a generator G, and a critic C, facing each other in a minmax game

$$\min_G \max_C \mathbb{E}_{x \sim P_X}[C(x)] - \mathbb{E}_{z \sim P_Z}[C(G(z))] \quad (1)$$

where G takes an input z from a latent space Z that follows a distribution P_Z , and creates counterfeit samples $G(z)$ that should emulate the real samples x from the real distribution P_X. Meanwhile, C examines both the real and counterfeit samples and outputs values $C(x)$ and $C(G(z))$ for each of them. C must maximize the difference between the scores of the real samples and the fakes, while G must reduce it. To accelerate convergence, a gradient penalty [66] term is added to the critic's objective function, which becomes

$$\mathbb{E}_{x \sim P_X}[C(x)] - \mathbb{E}_{z \sim P_Z}[C(G(z))] + \mathbb{E}_{g \sim P_G}[(\nabla_g ||g|| - 1)^2] \quad (2)$$

where P_G is defined uniformly sampling interpolations between sample pairs $(x, G(z))$.

Our model, which we named PowGAN, is a lighter version of the "Composer" model of the original MuseGAN that can generate piano rolls for drums, guitar, and bass (called a 'Power trio' in rock music, hence the name), taking Gaussian noise as input. The original MuseGAN model also included strings and piano, which we removed since we deemed they added too much complexity for humans (potentially non-experts) to evaluate within sensible time limits. The piano rolls used for training the model are a subset of the Lakh Pianoroll Dataset [40], which is derived from the Lakh MIDI Dataset [67].

Regarding the architecture, both the generator and critic are multi-stream 3D Convolutional Neural Networks. A schematic overview of the generator is depicted in Figure 2. The generator's architecture consists of an initial part shared among instruments that upsamples the input and later splits into private streams for each instrument. Each instrument stream performs inverse-3D convolutions over bars, time, and pitch, and is also split into two halves where dimension expansion occurs in different orders: time-first, and pitch-first. Each stream's halves are merged at the end to form the final track for each instrument. The critic follows an architecture mostly symmetric to the generator, with the addition of two global streams that capture harmony and rhythm features for all the composition [40].

Fig 2. PowGAN's generator architecture overview. The model takes a Gaussian noise vector, upsamples it through the shared network, and creates common features that are replicated for all instruments. Features run through private parallel instrument streams. Each instrument stream (drums, guitar, and bass) splits in two, upsamples these features, and merges again, each creating the bars for the corresponding track. Bars in each track are concatenated and form the final piano roll Ψ.

The features of the generated pieces, and the adequacy of the distribution and the model, are profiled through the following metrics:

- Empty Bar Ratio (EBR). Measures the percentage of completely silent bars. It ranges from 0 to 1. Applies to all tracks.
- Used Pitch Classes (UPC). Measures the number of different notes used in each pitched track. Classes circle back every scale, so the same note played in different scales counts as only one note. It ranges from 0 to 12. Applies to Guitar and Bass.
- Tonal Distance [68] (TD). Measures harmonic relations between pitched tracks. Intuitively, the lower the TD, the stronger the relation and the better the tracks sound together. Applies to the Guitar-Bass pair.

Bayesian optimization

admittedly, the math is not my strong suit

Bayesian optimization is our tool of choice for exploring the latent space of the developed model. BO seeks to optimize an unknown function f using a probabilistic surrogate model $P(f)$, commonly a Gaussian process (GP), and an acquisition function.

A Gaussian process is a collection of random variables such that every finite subset of them has a multivariate normal distribution, and it is defined by its mean $m(\cdot)$ and covariance $k(\cdot,\cdot)$ functions. The GP is built on a dataset $\mathcal{D}_n$ of n previous observations $(\mathbf{x}_i, y_i)$ of f, and its posterior probability $P(f|D)$ is updated every time a new observation is acquired. The posterior model allows predictions y_q at query points $\mathbf{x}_q$ which follow a normal distribution $y_q \sim \mathcal{N}(\mu(\mathbf{x}_q), \sigma^2(\mathbf{x}_q))$ such that

$$\begin{aligned}\mu(\mathbf{x}_q) &= \mathbf{k}^T(\mathbf{K} + \mathbf{I}\sigma_n^2)^{-1}\mathbf{y} \\ \sigma^2(\mathbf{x}_q) &= k(\mathbf{x}_q, \mathbf{x}_q) - \mathbf{k}^T(\mathbf{K} + \mathbf{I}\sigma_n^2)^{-1}\mathbf{k}\end{aligned} \tag{3}$$

where $\mathbf{y}$ is the vector of evaluation results, $\mathbf{k}$ is a vector with components $k_i = k(\mathbf{x}_q, \mathbf{x}_i)$, $\mathbf{K}$ is the kernel matrix with components $K_{ij} = k(\mathbf{x}_i, \mathbf{x}_j)$ for $i, j \in \{1, \ldots, n\}$ and σ_n^2 is the noise variance. There exist multiple options for $k(\cdot,\cdot)$ but the most extended practice is to use a stationary kernel, such as our choice for this work: the Automatic Relevance Determination (ARD) Matérn $\frac{5}{2}$ kernel with

$$\begin{aligned}k_{5/2}(\mathbf{x}, \mathbf{x}'|\boldsymbol{\theta}) &= \sigma_n^2\left(1 + \sqrt{5}r + \frac{5}{3}r^2\right)\exp\left(-\sqrt{5}r\right) \\ \mathbf{r}^2 &= (\mathbf{x} - \mathbf{x}')^T\boldsymbol{\Lambda}(\mathbf{x} - \mathbf{x}') \\ \boldsymbol{\Lambda} &= \mathrm{diag}(\boldsymbol{\theta}^{-1})\end{aligned} \tag{4}$$

where $\boldsymbol{\theta}$ are the lengthscales of the kernel, which measure correlation. In ARD kernels, each dimension has a different lengthscale. The larger the lengthscale, the less the function varies in that dimension, and the less relevant it is. These lengthscales are usually obtained through an *empirical Bayesian* approach, meaning they are estimated from either maximum likelihood or maximum a posteriori. These methods, however, result in an overconfident uncertainty estimation of the GP. We instead obtain the lengthscales through a *fully Bayesian* approach based on Markov chain Monte Carlo (MCMC) that trades higher computational cost for increased robustness. Since we work with human judgment, kernel computation is not a bottleneck in our procedure, so we can use it with almost no setbacks.

overconfident uncertainty – talk about an oxymoron ☺

In addition to the surrogate model, BO also uses an acquisition function $a(\mathbf{x}, P(f))$ for efficiently choosing the next evaluation point $\mathbf{x}_{t+1}$ at each step t of the algorithm, such that $\mathbf{x}_{t+1} = \mathrm{argmax}_{\mathbf{x}} a(\mathbf{x}, P(f|\mathcal{D}_t))$. Our choice for $a(\mathbf{x}, P(f))$ is the Expected Improvement (EI) function

$$\begin{aligned}\mathrm{EI}(\mathbf{x_q}) = \mathbb{E}_{p(y_q|\mathbf{x_q},\theta)}[\mathrm{I}(\mathbf{x_q})] &= (\rho - \mu(x_q))\Phi(z) + \sigma(x_q)\phi(z) \\ z &= (\rho - \mu(x_q))/\sigma(x_q)\end{aligned} \tag{5}$$

where $\mathrm{I}(\mathbf{x_q}) = \max(0, \rho - f(\mathbf{x_q}))$ is the improvement function, ρ is the current best result among the observations of the GP, ϕ and Φ are the Gaussian probability density function (PDF) and cumulative density function (CDF), and $\mu(x)$ and $\sigma(x)$ are obtained from Equation 3.

A representative initial dataset is key to fitting a meaningful initial model. We obtain our initial query points through Sobol sampling [69], a quasi-random series that ensures a good cover of the domain. After the initial evaluations, the algorithm runs iteratively by fitting the GP to all available observations and selecting new query points at the maxima of the acquisition function, as portrayed in Figure 3, adding the observations to the dataset. A pseudocode overview is provided in Algorithm 1.

Algorithm 1 Bayesian Optimization

Require: Objective function f, acquisition function a, budget N

Initialize data set $\mathcal{D} = (\boldsymbol{X}, \boldsymbol{y})$ with the data points $\boldsymbol{X} = \{x_1, x_2, \ldots, x_I\}$ and their responses $\boldsymbol{y} = \{y_1, y_2, \ldots, y_I\}$, using a preliminary design.
for $t = 1, 2, \ldots, N$ **do**
    Fit the surrogate model to $\mathcal{D}$: $P(f|\mathbf{X}, \mathbf{y}) \propto P(\mathbf{X}, \mathbf{y}|f)P(f)$
    $\mathbf{x}_t = \arg\max_x C(x|P(f|\mathbf{X}, \mathbf{y}))$
    $y_t = f(\mathbf{x}_t) + \sigma_n$ // With σ_n observation noise
    $\mathcal{D} = \mathcal{D} \cup (x_t, y_t)$
end for
Return $\arg\max_x(y|(x, y) \in \mathcal{D})$

Fig 3. Bayesian optimization overview. The GP (mean in blue, variance in pink) is fit to all previous observations (red dots) that follow the target function (dashed line) and the acquisition function a (EI, in green) indicates where the next query will be sampled.

Gaussian spaces

In order to explore a Gaussian space through BO, we need to adapt it from its assumed box-bounded search space into a Gaussian one. Some works [70] extend BO to iteratively increase the bounded volume, potentially to infinity, though it is still a hypercube. In our Gaussian latent space, the distribution of the data is concentrated around the origin, which means our search should be more thorough around this center area, as we can expect greater variation.

This sentence construction feels awkward with the additional clause regarding "greater variation."

To solve this, we use the Box-Muller (BM) transform to map an exploration space $[0, 1]^n$ to a Gaussian one $\mathcal{N}(\mathbf{0}, \mathbf{I}^n)$. We show an example of how this transformation affects a two-dimensional space in Figure 4, though it works for any even n number of dimensions.

Fig 4. Two-dimensional example of the Box-Muller transform. The Sobol-sampled data (left) ends up following a Gaussian distribution (right) after being transformed. This allows Bayesian optimization to explore Gaussian spaces while respecting the prior of the distribution.

High-dimensional Bayesian optimization

PowGAN's 256-dimensional latent space can be considered a high-dimensional space for performing global optimization. General non-linear optimization in high-dimensional

problems is an open problem. Most strategies assume that the optimization space can be simplified in a way such that the optimization can be performed in a simpler or lower-dimensional space. For example, some authors [23, 24, 64] assume that the optimization space has some additive structure $f(x_1, x_2, \ldots) = f(x_1) + f(x_2) + f(\ldots)$. Others perform only optimization in a local region [25] or assume the optimizer can be found in a lower-dimensional embedding [21, 62, 71]. For our setup, we hypothesize that a user's preferred sample can be found in a lower dimensional embedding S^d compared to the model latent space S^D, with dimensionality $d \ll D$, considering that some variables might be imperceptible for users not proficient in music or that certain regions of the search space might not capture harmony or proper musical structure.

In this section, we present a couple of baselines for dimensionality reduction along with *random rotational embedding Bayesian optimization* (ROMBO), our proposed method for high-dimensional optimization. For the baselines, we first build a nonlinear decoder from S^d to S^D by training a variational autoencoder on top of the PowGAN model, which preserves the Gaussian distribution of the latent space. However, that increases the complexity of the model and the training process. Second, we use *random embedding Bayesian optimization* (REMBO) [21] as a baseline for high-dimensional Bayesian optimization. REMBO also builds a decoder to enable sampling on a lower-dimensional embedding, which in this case is a random linear projection. This does not require extra training and guarantees finding a valid optimizer for the high-dimensional problem under certain conditions. REMBO is a fast and efficient method, but once again assumes a box-bounded input space, which conflicts with our Gaussian latent space. To solve this, we lastly introduce our REMBO-inspired algorithm, which we call *rotational random embedding Bayesian optimization* (ROMBO). The main advantages of ROMBO are its suitability for latent spaces with rotational symmetries, such as the Gaussian latent space of a GAN, and that its low-dimensional space bounds are simpler and fitter while still guaranteeing to find the optimum.

Neural decoder

As a baseline to create a decoder from the low-dimensional space to the high-dimensional space, we propose a neural decoder extending the generator architecture of our music generation model following a *variational autoencoder* (VAE) methodology.

An autoencoder is composed of two networks: an encoder E that reduces its input to fewer-dimensional features and a decoder D that must reconstruct the original data from these features. Variational autoencoders extend this by turning the latent encoded feature points into latent distributions by imposing a Gaussian prior. This ultimately forms a Gaussian latent space which can later be sampled. This new, smaller latent space and the following layers that upsample it back to a piano roll make up our baseline. In our proposal, we salvage a pre-trained PowGAN and use the generator as D; the critic, sans the head, as E; and place a fully connected core that connects them and learns the latent space, including mean and standard deviation layers to enforce the Gaussian prior. In order to maintain the expressiveness of the GAN, we freeze the layers of PowGAN's generator and train just the encoder and core of the model. This is portrayed in Figure 5. At the end of training, we keep the neural encoder that has learned to map the feature space S_d to PowGAN's latent space S_D.

In contrast to a GAN, where the two networks compete with each other, here E and D must both minimize a reconstruction loss $\mathcal{L}_R(x, D(E(x)))$. Common choices are the mean absolute error (MAE) and mean square error (MSE). However, these reconstruction errors are unfit for unbalanced, sparse, and heavily structured data, such as our piano rolls, where most values are silence and notes must follow specific patterns. We explore two alternatives for our training losses:

Fig 5. Variational-autoencoder-inspired architecture. We use PowGAN's beheaded critic C as encoder E, and generator G as decoder D. Features obtained through E are mapped to a low-dimensional Gaussian latent space (S_d) through fully connected layers. The feature vectors are then mapped back to PowGAN's high-dimensional latent space (S_D) and restored into piano rolls. All parts but PowGAN's generator are trained.

Asymmetrical loss [72]. The first one is an asymmetrical loss ($\mathcal{L}_{AS}$), which focuses on element-wise correspondence while trying to compensate for note/silence imbalance, defined as

$$\begin{aligned}\mathcal{L}_{AS}(\Psi, \Psi') &= \sum_{i,b,t,p}^{I,B,T,P} \psi_{ibtp}\mathcal{L}_{+}(\psi'_{ibtp}) + (1 - \psi_{ibtp})\mathcal{L}_{-}(\psi'_{ibtp}) \\ \mathcal{L}_{+}(x) &= (1 - x)^{\gamma_{+}} \log(x) \\ \mathcal{L}_{-}(x) &= (x_m)^{\gamma_{-}} \log(x - x_m) \\ x_m &= \max(x - m, 0)\end{aligned} \tag{6}$$

where Ψ and $\Psi' = D(E(\Psi))$ are the original and decoded piano rolls and ψ_{ibtp} is the element at track i, bar b, timestep t and pitch p of a piano roll Ψ. m is a negative clipping parameter, with values lower than m being set to zero. γ_{-} and γ_{+}, with $\gamma_{-} > \gamma_{+}$, are the asymmetric focus parameters that control how much negative predictions affect positive values and *vice versa*. Intuitively, this loss reduces the reward for easy true negatives, which prevents local minima such as complete silence (all zeros).

Perceptual loss. Element-wise losses can still lead to reconstructions with small errors that, perceptually, sound very different. This issue is tackled by using perceptual losses, which compare features, rather than single elements. One such example in the image domain is the structural similarity index measure (SSIM) [73], which compares patterns in pixel intensities. Thus, we designed a perceptual loss based on the previously commented musical features.

We first modify the EBR, as it can only output multiples of $\frac{1}{B}$ within the $[0, 1]$ interval for a single composition. We substitute it for an alternative, Silence Ratio (SR), calculated as

$$\mathrm{SR}_i(\Psi) = 1 - \frac{1}{BTP} \sum_{b,t,p}^{B,T,P} \psi_{ibtp} \tag{7}$$

for each track i in a piano roll, which measures the percentage of silent elements rather than bars. Then, we formulate our musical feature loss as

$$\mathcal{L}_{MF}(\Psi, \Psi') = ||(\mathcal{F}_M(\Psi) - \mathcal{F}_M(\Psi')||_2 \tag{8}$$

where $\mathcal{F}_M$ is a vector composed of the z-scores of the features calculated over the training data distribution for each of them. This includes SR for all instruments and TD and UPC for bass and guitar.

KL divergence. Whether we use $\mathcal{L}_{AS}$ or $\mathcal{L}_{MF}$ as $\mathcal{L}_R$, a Kullback-Leibler divergence (KL) term is always added to the loss to enforce a latent standard Gaussian distribution. The final loss of the architecture is

$$\mathcal{L} = \mathcal{L}_R(\Psi, \Psi') + \beta * \mathrm{KL}(P_V||\mathcal{N}(\mathbf{0}, \mathbf{I_d})) \tag{9}$$

where P_V is the latent space distribution, $\mathcal{N}(\mathbf{0}, \mathbf{I_d})$ is a d-dimensional standard Gaussian, and β is a weighting term, which increases and resets regularly by applying KL annealing [74]. This practice prevents the model from ignoring the reconstruction error in favor of minimizing KL divergence, especially at the early stages of training.

"Schroëdinger's symphony?" :)

Random Embedding Bayesian Optimization

Random Embedding Bayesian Optimization (REMBO) [21] works under the assumption that, for certain problems, most dimensions do not change the objective function. That is, the effective dimensionality of the problem is smaller than its input domain. One such example is shown in Figure 6, where the objective function is represented as a 2D function where there is only one important dimension, but we do not know which dimension is the important one at the beginning of optimization. Instead, we can build an embedded 1-dimensional subspace (represented as a red line in Figure 6) where we can perform optimization and we are guaranteed to find the optimum.

Fig 6. Example of a function over a 2-dimensional domain and one effective dimension. Searching over the 1-dimensional embedding is more efficient than searching over the whole domain.

Formally, a problem with low effective dimensionality is defined as the following:

Definition 1. [21] *A function $f : \mathbb{R}^D \rightarrow \mathbb{R}$ is said to have effective dimensionality d_e, with $d_e \leq D$, if*

- *there exists a linear subspace $\mathcal{T}$ of dimension d_e such that for all $\mathbf{x}_\top \in \mathcal{T} \subset \mathbb{R}^D$ and $\mathbf{x}_\perp \in \mathcal{T}^\perp \subset \mathbb{R}^D$, we have $f(\mathbf{x}_\top + \mathbf{x}_\perp) = f(\mathbf{x}_\top)$, where $\mathcal{T}^\perp$ denotes the orthogonal complement of $\mathcal{T}$.*
- *d_e is the smallest integer with this property.*

We call $\mathcal{T}$ the ***effective subspace*** *of f and $\mathcal{T}^\perp$ the* ***constant subspace****.*

REMBO works by building a random linear projection $\mathbf{A} \in \mathbb{R}^{Dxd}$, where the elements of $\mathbf{A}$ are independently sampled from a standard normal distribution $\mathcal{N}(0, 1)$. Assuming that $d \geq d_e$, Theorem 2 of Wang et al. [21] shows that for any point in the original input space $\mathbf{x} \in \mathbb{R}^D$ there exists a point $\mathbf{y} \in \mathbb{R}^d$, such that $f(\mathbf{x}) = f(\mathbf{Ay})$. Therefore, for any optimizer $\mathbf{x}^*$ there exists a point $\mathbf{y}^*$ such that $f(\mathbf{x}^*) = f(\mathbf{Ay}^*)$.

A limitation of REMBO is how to define the search space for the lower-dimensional space to avoid missing any point. The authors show that, for any origin-centered box constraint space $\mathcal{X} \subset \mathbb{R}^D$ where $\mathcal{X}$ is rescaled to $[-1, 1]^D$, and an effective dimensionality of d_e, then $||\mathbf{y}^*||_2 \leq \frac{\sqrt{d_e}}{\epsilon}||\mathbf{x}^*_\top||_2 \leq \frac{\sqrt{d_e}}{\epsilon}\sqrt{d_e}$ with probability at least $1 - \epsilon$ [21]. In practice, the effective dimensionality d_e is unknown, and we can only assume a dimension $d > d_e$. After empirical testing, they find $[-\sqrt{d}, \sqrt{d}]^d$ a suitable bound for the lower-dimensional space [21] which makes $\epsilon = \log(d)/\sqrt{d}$.

Our problem differs in that we want to find an optimizer $\mathbf{z}^*$ in a high-dimensional space S^D with a standard Gaussian prior $\mathcal{N}(\mathbf{0}, \mathbf{I}^D)$. To take into account this prior, we would want our embedding $\mathbf{A}$ and the samples $\mathbf{y} \in S^d$ to ensure that $\mathbf{Ay}$ follows a multivariate Gaussian distribution and that its confidence bounds match those of S^D. We show how achieving this with REMBO and BM alone is not possible, and provide graphic examples in Figure 7 of their results and the desired embeddings, which we obtain through our own adapted method proposed below.

REMBO(BM(y)) (Figure 7, left). Assuming our $\mathbf{y}$ is bounded within $[0, 1]^d$, this method results in REMBO projecting the points into d-dimensional Gaussians in S^D. This satisfies the multivariate Gaussian requirement. However, due to the random scaling of the embedding, the distribution does not necessarily match the confidence bounds of S^D.

Fig 7. Example embedding strategies. The 1D data is embedded in a 2D space, where the 95% confidence bound of $\mathcal{N}(0,1)$ is drawn in magenta. From left to right, data is embedded using REMBO on uniform data, REMBO on Gaussian data, Box-Muller after REMBO on uniform data, and our proposed method. The first two present scaling issues, and the third creates spiral patterns with undesirable artifacts. Our method embeds data as multivariate Gaussians with coincident confidence bounds with the standard distribution.

BM(REMBO(y)) (Figure 7, middle) Embedding data within the interval $[-\sqrt{d}, \sqrt{d}]^d$ and applying the box constraint, as proposed in REMBO, projects $\mathbf{Ay}$ within $[-1,1]^D$. Re-scaling them to $(0,1]$ and applying Box-Muller forms spiral-like patterns. They provide no guarantee regarding the distribution of the data nor the confidence bounds, and sometimes create artifacts due to the box-constraint clipping, with many points being mapped either to infinity ($\ln(\epsilon)$ with some very small positive ϵ) or the origin.

Since none of these approaches fulfill the requirements, we propose a novel methodology (Figure 7, right) that adapts REMBO to work as intended in Gaussian spaces.

Random rotational embedding Bayesian optimization

Below, we explain how to adapt REMBO to improve its performance when working with Gaussian spaces and how to overcome some of its assumptions, like the box constraint.

Our proposed algorithm, which we call *random rotational embedding Bayesian optimization* (ROMBO), solves the scaling issue by redefining the embedding matrix so it acts as a rotation rather than a random projection. We provide our process for obtaining this new embedding and proof of its effectiveness below.

Theorem 2. *(based on Theorem 2 from Wang et al. [21]) Assume we are given a function $f : \mathbb{R}^D \to \mathbb{R}$ with effective dimensionality d_e and a matrix $\mathbf{Q} \in \mathbb{R}^{D\times d}$, with $d \geq d_e$, whose columns form a random orthonormal basis for some d-dimensional subspace $\mathcal{Q} \subset \mathbb{R}^D$. Then, with probability 1, for any $\mathbf{z} \in \mathbb{R}^D$ there exists a $\mathbf{v} \in \mathbb{R}^d$ such that $f(\mathbf{z}) = f(\mathbf{Qv})$.*

Proof. Following Wang et al. [21], we just need to prove that the matrix $\mathbf{\Phi}^T\mathbf{Q}$, where $\mathbf{\Phi} \in \mathbb{R}^{D\times d_e}$ is an orthonormal basis for $\mathcal{T}$, has rank d_e. The rest of the proof applies for any matrix $\mathbf{Q}$ that satisfies that. Both $\mathbf{\Phi}$ and $\mathbf{Q}$ have orthonormal columns, and hence are full rank d_e and d, respectively. Their product $\mathbf{\Phi}^T\mathbf{Q}$ is a $d_e \times d$ matrix, with rank at most d_e. Since $\mathbf{Q}$ is randomly oriented relative to $\mathbf{\Phi}$, $\mathbf{\Phi}^T\mathbf{Q}$ will have rank d_e with probability 1. □

For our algorithm, we obtain $\mathbf{Q}$ from the *QR-decomposition* of $\mathbf{A}$, such that $\mathbf{A} = \mathbf{QR}$. We use the same strategy as REMBO and use a random $\mathbf{A}$ matrix whose entries are independent normal samples from $\mathcal{N}(0,1)$. Then, as per the results from Steward [75], we can see that for any matrix $\mathbf{A}$ whose elements are independently normally distributed with mean zero and common variance σ^2, then $\mathbf{Q}$ is an orthonormal basis with random orientation. Although the result from Steward [75] is defined for full rank matrices $\mathbf{A_s} \in \mathbb{R}^{D\times D}$, with $\mathbf{A_s} = \mathbf{Q_s}\mathbf{R_s}$, we can see that our matrix $\mathbf{Q}$ is just the first d columns of $\mathbf{Q_s}$ representing the orthogonal basis for the column space of $\mathbf{A}$. Thus, if $\mathbf{Q_s}$ has random orientation, then $\mathbf{Q}$ has random orientation as well in the column space of $\mathbf{A}$.

Next, we need to consider the optimization problem in a compact space $\mathcal{Z} \in \mathbb{R}^D$ and how we can define the bounds in the embedding $\mathcal{V} \in \mathbb{R}^d$, which, as discussed before, is one of the limitations of REMBO.

Theorem 3. *(based on Theorem 3 from Wang et al. [21]) Suppose we want to optimize a function $f : \mathbb{R}^D \rightarrow \mathbb{R}$ with effective dimension $d^e \leq d$. Suppose further that the effective subspace $\mathcal{T}$ of f is such that $\mathcal{T}$ is the span of d_e basis vectors, and let $\mathbf{z}^*_\top \in \mathcal{T}$ be an optimizer of f inside $\mathcal{T}$. If $\mathbf{Q}$ is a $D \times d$ matrix such that its columns form a random orthonormal basis for $\mathbb{R}^d$, there exists an optimizer $\mathbf{v}^* \in \mathbb{R}^d$ such that $f(\mathbf{Qv}^*) = f(\mathbf{z}^*_\top)$ and $||\mathbf{v}^*||_2 = ||\mathbf{z}^*_\top||_2$.*

Proof. In this case, our proof is simpler than Theorem 3 [21]. Since $\mathbf{Q}$ is an orthonormal basis, $\mathbf{Qv}^*$ is a rotation of $\mathbf{v}^*$ in S^D, as all d singular values of $\mathbf{Q}$ are 1. Therefore, $||\mathbf{v}^*||_2 = ||\mathbf{Qv}^*||_2 = ||\mathbf{z}^*||_2$. □

This result is quite important. Contrary to REMBO, our projection matrix $\mathbf{Q}$ does not change the size of our exploration space. This is intuitive when we consider that REMBO builds its input space in a hypercube, while our input space is a hypersphere thanks to the rotational symmetry of the latent space as a Gaussian distribution, and the Box-Muller transformation. Therefore, we can maintain the same constant bounds independently of the effective dimension, and therefore, we can find tight bounds even if the effective dimension is unknown. As shown in Figure 7, our method preserves the bounds of the search and input spaces.

Pipeline evaluation

Music generation model

The model architecture follows the schema displayed in Figure 2. As discussed, our final piano rolls have three instruments instead of five. The temporal resolution of our output is also coarser, going down from 48 timesteps per bar to 16. The final output of our model is a piano roll $\Psi \in [0, 1]^{3\times4\times16\times72}$. We provide a layer breakdown of the generator in Table 1.

	Kernel	Stride	Output	Channels
**Shared network**	(4,1,1)	(4,1,1)	(4,1,1)	256
	(1,4,3)	(1,4,3)	(4,4,3)	128
	(1,2,3)	(1,2,2)	(4,8,6)	64
**Private pitch-first**	(1,1,12)	(1,1,12)	(4,8,72)	16
	(1,2,1)	(1,2,1)	(4,16,72)	1
**Private time-first**	(1,2,1)	(1,2,1)	(4,16,6)	16
	(1,1,12)	(1,1,12)	(4,16,72)	1
**Private merge**	(1,1,1)	(1,1,1)	(4,16,72)	1

Table 1. Layer breakdown of PowGAN's generator. 'Shared' layers are common for all instruments, while 'Private' ones are replicated per instrument. Private pitch-first and time-first layers are parallel, and they end up merging with a 1x1 convolution.

For the training data, each song is divided into 4-bar fragments, which are then sampled uniformly up to a maximum of 18 fragments per song. We ensure that removing the piano and strings does not accidentally create silent fragments, though we maintain them if they were already silent, even with all instruments. Our final training dataset is made of 57.201 fragments. For evaluation, we compare the musical features of the generated songs and the dataset used for training. We measure the EBR, UPC, and TD of a new dataset of 64000 fragments (2000 batches of 32) generated by sampling PowGAN's high-dimensional Gaussian latent space. We report the metrics obtained by

57 Thousand? fragments?

the PowGAN generations in Table 2, and provide the ones reported by MuseGAN [40] as reference. The overall absolute error between our dataset and our model for each of these metrics is similar in scale to the errors reported for MuseGAN, and even improves them in the case of UPCs and EBR for the drum track. With these results, we deem the training as correct and use PowGAN as our music generation model for the optimization tests.

		EBR (%)			UPC		TD
		D	G	B	G	B	G-B
**MuseGAN**	Dataset	8.06	19.4	8.06	3.08	1.71	1.57
	Model	2.33	18.3	6.59	3.69	1.53	1.56
	Abs. Error	5.73	1.10	1.47	0.61	0.18	0.01
**PowGAN**	Dataset	6.40	20.07	8.74	3.43	2.25	1.27
	Model	8.50	18.75	6.53	3.36	2.14	1.32
	Abs. Error	2.10	1.32	2.21	0.07	0.11	0.05

Table 2. Metric comparison between the used dataset and the closest values reported in evaluation, both for the MuseGAN baseline and PowGAN. Values in the 'Model' row are better the closer to the 'Dataset' row. Notice that both datasets show different metrics due to random sampling, track trimming, and sample rejection, but the error still shows a similar scale, which suggests correct training. For the columns, 'D', 'B', and 'G' correspond to Drums, Bass, and Guitar.

The code for training and evaluation was implemented using PyTorch and based on MuseGAN's implementation. We train the model for 25000 steps, with a batch size of 32 and using an Adam optimizer with $\beta_1 = 0.6$ and $\beta_2 = 0.9$ and otherwise PyTorch default parameters. The generator and critic each use a different optimizer, and at each step, the critic performs 5 forward passes for every forward pass of the generator. For the first 10 steps, these passes are increased to 50, to give the critic a head start. The training was performed on a free Google Colaboratory environment equipped with an Intel Xeon CPU with 2 vCPUs and 13GB of RAM, as well as an NVIDIA Tesla T4 GPU with 16GB of VRAM. The whole execution of the notebook takes about an hour to complete under these conditions.

High-dimensional optimization

The neural decoders are trained using the dataset generated with PowGAN, which is split in a 90/10 ratio for the train and test sets. We use this and not PowGAN's training data because a GAN generator tends not to replicate the individual data points used to train it [76]. Our decoder is a frozen pre-trained generator, so we cannot confirm whether it can generate the exact pieces used to train PowGAN or not. With a training set made of its generations, we ensure the decoder can replicate them once again.

We use both $\mathcal{L}_{AS}$ and $\mathcal{L}_{MF}$ to train two neural decoders, referred as ND_{AS} and ND_{MF} below. The training was performed on a 12th Gen Intel Core™ i7-12700K CPU with 32GB of RAM and an NVIDIA GeForce RTX 4090 GPU with 24GB of VRAM. We train for 10 epochs and a batch size of 32. The execution takes between fifteen and thirty minutes under these conditions, depending on the loss used for training. REMBO and ROMBO do not need additional training, as discussed earlier. To run the experiments, we develop an application based on the BO library BayesOpt [77]. We use the Python interface for BayesOpt, which provides a wrapper for custom optimization problems.

We design a series of experiments to test the efficacy of the trained neural decoders, REMBO, and ROMBO. The objective of each experiment is to recreate a song

generated by forward-passing a point randomly sampled from S^D through the PowGAN generator. This song represents a hypothetical subject's favorite song, which acts as the target. A total of 20 targets are generated, which are the same for all decoders.

Our objective function starts taking a query point from our $S^d = [0,1]^d$. For REMBO, it rescales it to the recommended $[-\sqrt{d}, \sqrt{d}]^d$ interval. For the others, it performs the Box-Muller transform into $\mathcal{N}(\mathbf{0}, \mathbf{I}^d)$. The resultant query is projected onto S^D through the selected decoder, and the generator produces the resultant song. The loss (either $\mathcal{L}_{AS}$ or $\mathcal{L}_{MF}$) between this song and the target is the function output. For $\mathcal{L}_{AS}$ we chose $\gamma_+ = 4, \gamma_- = 16$ and $m = 0.05$ as our hyperparameters.

We select $d = 10$ for our latent space dimensionality. All experiments use the 5/2 Matèrn kernel with ARD and the Expected Improvement acquisition function. For REMBO and ROMBO, all trials were done with a single random embedding per target, and the embedding for ROMBO was the $\mathbf{Q}$ matrix obtained from the QR-decomposition of the $\mathbf{A}$ matrix used for REMBO for that same target. We start each trial with Sobol sampling, collecting 10 data points, and the optimization budget after initialization is set to 60 samples.

The results of the automatic trials, collected in Figures 8 and 9 Table 3, show the evolution and final average values of each loss function through optimization.

Fig 8. Average historic best (with 95% confidence bounds) showcasing the evolution of $\mathcal{L}_{MF}$ (top) and $\mathcal{L}_{AS}$ (bottom) during optimization of $\mathcal{L}_{MF}$ (left) and $\mathcal{L}_{AS}$ (right) for both REMBO (red) and ROMBO (green). ROMBO outperforms REMBO and both NDs in all metrics and targets.

Fig 9. Average historic best (with 95% confidence bounds, plotted in logscale) showcasing the evolution of $\mathcal{L}_{MF}$ (top) and $\mathcal{L}_{AS}$ (bottom) during optimization of $\mathcal{L}_{MF}$ (left) and $\mathcal{L}_{AS}$ (right) for ROMBO (green) and the NDs trained minimizing $\mathcal{L}_{MF}$ (yellow) and $\mathcal{L}_{AS}$ (blue).

		**Obtained metric**	
**Optimizing:**	**With decoder:**	$\mathcal{L}_{MF}$	$\mathcal{L}_{AS}$
$\mathcal{L}_{MF}$	ND_{MF}	2.8240	1.1502
	ND_{AS}	1.6969	0.9410
	REMBO	0.5728	0.9185
	ROMBO	**0.4397**	**0.7762**
$\mathcal{L}_{AS}$	ND_{MF}	2.9550	1.1400
	ND_{AS}	1.8268	0.9488
	REMBO	0.7437	0.9010
	ROMBO	**0.5164**	**0.7444**

Table 3. Final average loss after optimization for each decoder and loss combination. ROMBO outperforms REMBO and both NDs in both metrics when optimizing either of the two loss functions.

ROMBO outperforms, on average, both REMBO and the neural encoders. This is true for both $\mathcal{L}_{AS}$ and $\mathcal{L}_{MF}$ even when optimizing with a metric and evaluating the other. ROMBO shows an early advantage that is maintained throughout optimization, suggesting better exploration, as intended when formulating the method.

The curves of the neural decoders also offer additional insight into the neural encoders' behavior. Despite being trained to minimize their respective losses, the one

trained on $\mathcal{L}_{MF}$ is significantly worse at both. This may be due to the model itself not being complex enough or the loss not being fit for gradient-descent-based strategies. As for the ND_{AS}, its performance is closer to ROMBO, though it still doesn't reach the same values, and the latter has less variance in the results. Both metrics follow similar curves during optimization, with slight improvements for the optimized metric at each run. These results show that ROMBO is the best method out of those we could develop and are proof of our method's effectiveness. All further tests are carried out using ROMBO as our decoder.

Experiment Design and Procedure

We conclude our work with a human-driven test.

Experimental design

We switch our evaluation method to human judgment, a numerical evaluation from 0 to 10, and they are asked to try and find a favorite generation. We recruited participants for our experiment for a total of two weeks (February 1st to 15th of 2025). A total of sixteen people enrolled, four female and twelve male participants between the ages of 23 and 43, who are divided into two groups of eight (from now the 'Bayesopt' and 'Random' groups). The first group uses the whole pipeline with ROMBO, and the second listens to random samples from PowGAN. Participants are not told this information. The objective is to evaluate the optimization's effectiveness compared to randomly asking the model to generate pieces and to see whether users think the sample refinement process is useful, regardless of the selected method.

Ethics statement

The research process was conducted according to the ethical recommendations of the Declaration of Helsinki and was approved by both our institution's data protection unit and the Aragón Autonomous Community Research Ethics Committee (CEICA), which supervises fairness in research projects and the use of biological and personal data. Our protocol is non-invasive and involves the gathering of opinion data only, with no risk for any participant, who gave their informed written consent right after explaining the experiment. The consent allowed the abandonment of the experiment, and all data was analyzed anonymously.

Procedure

After collecting the volunteer's consent, they are told the following pieces of information:

- They will be listening to several computer-generated, 10-second-long musical compositions. The objective is to find the song that is best suited to their taste among the possible generations.
- They will be giving a score to each of the compositions, from 0 to 10, and with 0.1 precision. Scores are said aloud to the researcher. Once they confirm their decision, the scores can't be changed. If they give the same highest score twice, the latter will be considered better.
- They can ask the supervisor to replay each composition once, before giving the score, if they need to. They can also ask to replay the one they gave the current best score to and be reminded of what the score was.

- The experiment lasts for approximately 30 minutes, accounting for short breaks every few songs. The supervisor will tell them when a break starts and stops.

Then, the supervisor generates and plays two random songs. The user is told not to give a score for these songs. This serves two purposes: First, letting the participant know how the generated songs sound, to control expectations, and to give users a preamble to palliate common biases of opinion problems, such as drift and anchoring. Second, giving them the chance to adjust the volume. The user is intended to turn it up enough so they can clearly hear the music, but not so much as to be painful to hear.

We choose the experiments' parameters taking into account the limitations of real users, which mainly involve evaluation time, capping the initial dataset size and iteration budget to a 30-minute time limit. The final number of observations is 22 samples using Sobol and 42 samples using optimization, for a total of 64 samples. Compared to the automatic trials (10 Sobol samples and 60 acquired samples), this gives volunteers more chances to hear different kinds of compositions, potentially finding one that caters to their taste. It is also a way to prepare for noisy observations, as automatic trials used an exact loss, but we cannot expect the same for human evaluation. The breaks are distributed uniformly along the duration of the experiment, with two thirty-second breaks when reaching 16 and 48 evaluations, and a one-minute break when reaching 32 of them. These breaks both reduce the possible fatigue of the user and reset the hearing, so evaluations are not that conditioned on how long users have been listening to music without stopping.

Then, after completing the optimization process, volunteers are introduced to a survey stating four affirmations about the model's general performance and the optimization process. All statements can be answered using Likert scale answers: "Strongly disagree", "Disagree", "Neither agree nor disagree" (onward "No preference", for short), "Agree", and "Strongly agree". Users are asked to tell how much they agree with the following points:

- Q1: "Overall, I like the generated songs"
- Q2: "I like the song I gave the best score to"
- Q3: "I like the song I gave the best score to better than the first song I listened to"
- Q4: "After listening to all the songs, I am alright with the scores I gave to the best and first songs"

Question Q1 asks about the general performance of the composition model. Questions Q2 and Q3 evaluate the need for an optimization pipeline. Finally, question Q4 asks about the users' ability to act as judges, since they are not given a chance to correct themselves and may not agree with their own judgment after the experiment ends.

Results

For the response to the final survey, Figure 10 shows the distribution of answers for all 16 participants. Most participants answer either neutrally or negatively to Q1, meaning they do not explicitly like the generated songs in general. However, both groups agree that they like their final selected piece and most times they like it better than the first one they heard (Q2 and Q3), meaning that users are not willing to settle for the first things they obtain from a generative model and appreciate customization of deep learning models to individual tastes. Finally, most users agree with the grades they

Fig 10. Aggregated column charts showcasing the results of the Likert survey. All questions show overall positive responses. Though most users do not like most of the pieces (Q1), they always like the one they graded best (Q2), in most cases better than the first one (Q3), and they are alright with their grades (Q4).

have given during the experiment (Q4), which supports our experimental procedure and our choice of scalar grades as a goodness function.

We next compare the musical features obtained by each of our volunteers' final pieces and the model's average metrics. For this analysis, we use the same metrics used to evaluate the training of the composition model: UPC for guitar and bass, and the TD between them. SR is omitted for brevity's sake, as it is stable across all generated compositions, with small variability in the whole latent space. Figure 11 and Tables 4 and 5 show a per-user display of the obtained metrics.

	Dataset avg.	Model avg.	Group avg.	S1	S2	S3	S4	S5	S6	S7	S8
**UPC (B)**	2.25	2.14	1.56	2.00	1.75	1.25	0.00	1.75	2.00	1.50	2.00
**UPC (G)**	3.43	3.36	4.25	4.75	5.00	3.25	0.00	4.75	5.25	3.75	7.25
**TD (B-G)**	1.27	1.32	1.35	1.12	1.45	1.69	N/A	1.39	1.29	1.25	1.23

Table 4. Metrics of the Bayesopt group participants' final selected songs. UPCs for guitar (G) and bass (B) are consistently above and below average, respectively, and TD is slightly higher.

	Dataset avg.	Model avg.	Group avg.	RS1	RS2	RS3	RS4	RS5	RS6	RS7	RS8
**UPC (B)**	2.25	2.14	3.34	3.25	3.25	4.50	3.75	1.75	4.5	1.75	1.50
**UPC (G)**	3.43	3.36	3.28	4.00	6.00	5.75	1.75	0.00	0.00	4.75	4.00
**TD (B-G)**	1.27	1.32	1.01	0.84	1.25	0.64	1.39	N/A	N/A	0.96	1.01

Table 5. Metrics of the Random group participants' final selected songs. The Guitar track reports an average UPC close to the model mean, but UPCs for the Bass track are greatly above average. TD is consistently lower than the model's average.

Fig 11. Per-user bar chart of UPCs and TD for each of the participants' best-graded compositions, and average results (with 95% confidence bounds) for the dataset, the model, and the Bayesopt and Random groups. The Bayesopt group shows a close TD and a good Guitar-Bass balance for UPCs, while the Random group tends to select songs with high UPC in the bass, hinting at exploration around irregular regions of the distribution.

Overall, the metrics show significant variations among different users. The Random group has a closer-to-average UPC for the guitar track, but much larger for the bass track. This also has an impact on the TD, as two harmonic tracks (both playing chords) tend to have lower distances than a harmonic track and a melody track (one playing chords and the other single notes), which is the common behavior of the guitar and bass tracks [40]. Considering that for the Random group exploration was not guided by feedback, unlike for the Bayesopt group, this may be an indicator of the optimization pipeline helping to avoid irregular regions of the latent space. Meanwhile, only one corner case is found among the Bayesopt group: subject 4. In their experiment, they gave high scores to compositions that silenced both the bass and guitar tracks, finding the optimum in a drums-only composition. The model's probability to do so on its own, without human guidance, lies at 0.9%. In this case, the irregular behavior was

Group		Bayesopt	Random	Difference
**# of updates (↑)**	**Avg.**	**7.00**	5.00	+2.00
**No-new-max streak (↓)**	**Avg.**	**8.29**	9.96	-1.67
	**Median**	**2.75**	3.93	-1.18
	**Longest**	**34.62**	36.50	-1.88
**Low-grade streak (↓)**	**Avg.**	**2.89**	3.52	-0.63
	**Median**	**2.19**	2.56	-0.37
	**Longest**	**7.75**	8.75	-1.00

Table 6. Metrics for both our Bayesopt and Random groups. All metrics are averaged over the subjects of each group. "No-new-max streaks" are periods without a new maximum in their grades. "Low-grade streaks" are periods with a 3-iteration rolling average below the mean of the maximum and minimum values up to the current iteration. The 'Difference' column shows how much the Bayesopt group metrics improve with respect to the Random group.

intentional, as the user rewarded the first drum solo they encountered, and the system started generating more until the end of the experiment.

As a final test with both groups, we compared the impact of Bayesian optimization during the duration of the experiment by looking at the grades' evolution with and without the optimization running. Our measurements focus on the experience of users when using the pipeline, emphasizing efficiency. Optimization should help users find preferred compositions faster and more often. Also, exploration should be guided and avoid uninteresting regions within possibility. Specifically, we retrieve the number of maximum score updates along the whole optimization and define two metrics: no-new-max streaks and low-grade streaks. No-new-max streaks are iterations in a row without a new maximum, whereas Low-grade streaks are periods where the average of the last three scores is below the average of the maximum and minimum values so far. The first one measures how often users update their best graded composition, and the second measures how long they spend giving sub-par grades in a row. The collected metrics are showcased in Table 6.

These metrics show how the users in the Bayesopt group have shorter no-new-max streaks and more best score updates overall. Their low-grade streaks are also shorter. This suggests that optimization may indeed help avoid uninteresting areas of the search space. The difference in best score updates stays consistent over the optimization process, as showcased in Figure 12, including the initial budget acquisition. This emphasizes the inefficiency of high-dimensional space exploration and suggests that ROMBO is an effective way to reduce dimensionality without hindering performance.

Fig 12. Evolution of the average maximum value update number (with 95% confidence bounds) throughout optimization for the Bayesopt and Random groups. The left side shows the experiment's setup, with equal values given later being considered new maxima. The right side shows evolution using strictly higher scores, for analysis completion. In both cases, the optimization group shows a consistent advantage.

After a qualitative analysis of the experiment results and the volunteers' behavior and comments throughout its duration, we gathered some additional findings. First, though they are allowed to replay the fragments, few of the participants used this functionality. Even at the final survey, when asked if they needed to replay the first fragment (which they listened to 30 minutes earlier), some said they remembered it. We take this as an indicator of memorization for comparison not being an issue, with early samples still providing information later on. Second, though allowed to use 0.1 precision, the general tendency is to use rounded grades or 0.5 precision. This particular behavior

is of special importance for the last part of the second clause of the experiment stated above ("if they give the same highest score twice, the latter will be considered better"), as it is something users exploit. If the grade difference between the best fragment candidates is small. instead of upping the ante by 0.1, most users' preferred solution is to keep the round score and override it. We make sure that this does not greatly impact our pipeline's performance, as Figure 12 shows, as even with strictly higher scores, our optimization group still obtains more updates for their best graded compositions. This may be due to users preferring to keep a reduced collection of distinct given scores to facilitate keeping track of the fragments they have already graded. Overall, results show that the optimization process helps sample better candidates than randomly querying the model, and users seem to find the pipeline useful and quickly gain proficiency with it.

Conclusion

We present random rotational embedding Bayesian optimization (ROMBO), a Bayesian optimization algorithm for high-dimensional optimization in Gaussian search spaces. We employ this algorithm in a system able to tune a music generation model to produce a favorite piece using human feedback given in real time. With a custom music generation model based on the gold-standard model MuseGAN: PowGAN, we are capable of generating piano rolls for drums, guitar and bass. We then optimize a target function mapping a point from its Gaussian latent space to a human-given grade for the resultant song. To our knowledge, this is the first work to apply Bayesian optimization for the complex case of several polyphonic instrument tracks.

In our automatic experiments, we show that ROMBO outperforms another high-dimensional Bayesian optimization baseline (REMBO), which assumes box-bounded search spaces, when optimizing over the Gaussian latent space of the model. This shows that, when working with these Gaussian high-dimensional spaces, it is important and beneficial to ensure the embedded data preserves the confidence bounds of the high-dimensional space distribution to achieve better results in fewer iterations.

Furthermore, our human-driven experiments reveal that Bayesian optimization, and ROMBO in particular, are effective methods to improve the experience of users when trying to obtain a desired sample from a music generation model. Comparing those using ROMBO and those querying the model as-is, ROMBO provides them with more potentially best-regarded pieces, and also guides exploration to avoid uninteresting regions of the latent space. This is of most importance, as our survey reveals that users are not willing to settle for the early results of a generative model, and regard positively a refinement stage, in which the guiding capabilities of ROMBO can be most useful.

References

1. Dhariwal P, Jun H, Payne C, Kim J, Radford A, Sutskever I. Jukebox: A Generative Model for Music; 2020.
2. Schumacher D, Labounty F. Enhancing BARK Text-to-Speech Model: Addressing Limitations through Meta's Encodec and Pretrained HuBert; 2023.
3. Défossez A, Copet J, Synnaeve G, Adi Y. High Fidelity Neural Audio Compression; 2022.

4. Kreuk F, Synnaeve G, Polyak A, Singer U, Défossez A, Copet J, et al. AudioGen: Textually Guided Audio Generation. In: The Eleventh International Conference on Learning Representations; 2023.

5. Copet J, Kreuk F, Gat I, Remez T, Kant D, Synnaeve G, et al. Simple and controllable music generation. Advances in Neural Information Processing Systems. 2024;36.

6. Dahms R, Eicher C, Haesner M, Mueller-Werdan U. Influence of music therapy and music-based interventions on dementia: A pilot study. Journal of Music Therapy. 2021;58(3):e12–e36.

7. Pelletier CL. The effect of music on decreasing arousal due to stress: A meta-analysis. Journal of music therapy. 2004;41(3):192–214.

8. Herremans D, Chuan CH, Chew E. A functional taxonomy of music generation systems. ACM Computing Surveys (CSUR). 2017;50(5):1–30.

9. Christiano PF, Leike J, Brown T, Martic M, Legg S, Amodei D. Deep reinforcement learning from human preferences. Advances in neural information processing systems. 2017;30.

10. Ouyang L, Wu J, Jiang X, Almeida D, Wainwright C, Mishkin P, et al. Training language models to follow instructions with human feedback. Advances in neural information processing systems. 2022;35:27730–27744.

11. Garnett R. Bayesian Optimization. Cambridge University Press; 2023.

12. Martinez-Cantin R. Funneled Bayesian optimization for design, tuning and control of autonomous systems. IEEE transactions on cybernetics. 2018;49(4):1489–1500.

13. Shahriari B, Swersky K, Wang Z, Adams RP, De Freitas N. Taking the human out of the loop: A review of Bayesian optimization. Proceedings of the IEEE. 2015;104(1):148–175.

14. Ding Y, Kim M, Kuindersma S, Walsh CJ. Human-in-the-loop optimization of hip assistance with a soft exosuit during walking. Science robotics. 2018;3(15):eaar5438.

15. Kim M, Ding Y, Malcolm P, Speeckaert J, Siviy CJ, Walsh CJ, et al. Human-in-the-loop Bayesian optimization of wearable device parameters. PloS one. 2017;12(9):e0184054.

16. Koyama Y, Sato I, Goto M. Sequential gallery for interactive visual design optimization. ACM Trans Graph. 2020;39(4). doi:10.1145/3386569.3392444.

17. Brochu E, Brochu T, De Freitas N. A Bayesian interactive optimization approach to procedural animation design. In: Proceedings of the 2010 ACM SIGGRAPH/Eurographics Symposium on Computer Animation; 2010. p. 103–112.

18. Brochu E, Freitas N, Ghosh A. Active preference learning with discrete choice data. Advances in neural information processing systems. 2007;20.

19. Martinez-Cantin R, Tee K, McCourt M. Practical Bayesian optimization in the presence of outliers. In: International conference on artificial intelligence and statistics. PMLR; 2018. p. 1722–1731.

20. Garcia-Barcos J, Martinez-Cantin R. Robust policy search for robot navigation. IEEE Robotics and Automation Letters. 2021;6(2):2389–2396.

21. Wang Z, Hutter F, Zoghi M, Matheson D, De Feitas N. Bayesian optimization in a billion dimensions via random embeddings. Journal of Artificial Intelligence Research. 2016;55:361–387.

22. Letham B, Calandra R, Rai A, Bakshy E. Re-examining linear embeddings for high-dimensional Bayesian optimization. Advances in neural information processing systems. 2020;33:1546–1558.

23. Kandasamy K, Schneider J, Póczos B. High dimensional Bayesian optimisation and bandits via additive models. In: International conference on machine learning. PMLR; 2015. p. 295–304.

24. Wang Z, Li C, Jegelka S, Kohli P. Batched high-dimensional Bayesian optimization via structural kernel learning. In: International conference on machine learning. PMLR; 2017. p. 3656–3664.

25. Eriksson D, Pearce M, Gardner J, Turner RD, Poloczek M. Scalable global optimization via local Bayesian optimization. Advances in neural information processing systems. 2019;32.

26. Zhou Y, Koyama Y, Goto M, Igarashi T. Interactive exploration-exploitation balancing for generative melody composition. In: Proceedings of the 26th International Conference on Intelligent User Interfaces; 2021. p. 43–47.

27. Goodfellow I, Pouget-Abadie J, Mirza M, Xu B, Warde-Farley D, Ozair S, et al. Generative adversarial networks. Communications of the ACM. 2020;63(11):139–144.

28. Arjovsky M, Chintala S, Bottou L. Wasserstein generative adversarial networks. In: International conference on machine learning. PMLR; 2017. p. 214–223.

29. Kingma DP, Welling M. Auto-Encoding Variational Bayes. In: 2nd International Conference on Learning Representations, ICLR 2014, Banff, AB, Canada, April 14-16, 2014, Conference Track Proceedings; 2014.

30. Rezende DJ, Mohamed S, Wierstra D. Stochastic backpropagation and approximate inference in deep generative models. In: International conference on machine learning. PMLR; 2014. p. 1278–1286.

31. Ho J, Jain A, Abbeel P. Denoising diffusion probabilistic models. Advances in neural information processing systems. 2020;33:6840–6851.

32. Yenduri G, Ramalingam M, Selvi GC, Supriya Y, Srivastava G, Maddikunta PKR, et al. Gpt (generative pre-trained transformer)–a comprehensive review on enabling technologies, potential applications, emerging challenges, and future directions. IEEE Access. 2024;.

33. Selivanov A, Rogov OY, Chesakŏv D, Shelmanov A, Fedulova I, Dylov DV. Medical image captioning via generative pretrained transformers. Scientific Reports. 2023;13(1):4171.

34. Vaswani A. Attention is all you need. Advances in Neural Information Processing Systems. 2017;.

35. Rafailov R, Sharma A, Mitchell E, Manning CD, Ermon S, Finn C. Direct preference optimization: Your language model is secretly a reward model. Advances in Neural Information Processing Systems. 2024:36.

36. Mozer MC. Neural Network Music Composition by Prediction: Exploring the Benefits of Psychoacoustic Constraints and Multi-scale Processing. In: Musical Networks: Parallel Distributed Perception and Performance; 1999.

37. Todd PM. A connectionist approach to algorithmic composition. Computer Music Journal. 1989;13(4):27–43.

38. Eck D, Schmidhuber J. Finding temporal structure in music: Blues improvisation with LSTM recurrent networks. In: Proceedings of the 12th IEEE workshop on neural networks for signal processing. IEEE; 2002. p. 747–756.

39. Hadjeres G, Pachet F, Nielsen F. Deepbach: a steerable model for bach chorales generation. In: International conference on machine learning. PMLR; 2017. p. 1362–1371.

40. Dong HW, Hsiao WY, Yang LC, Yang YH. Musegan: Multi-track sequential generative adversarial networks for symbolic music generation and accompaniment. In: Proceedings of the AAAI Conference on Artificial Intelligence. vol. 32; 2018.

41. Yu L, Zhang W, Wang J, Yu Y. Seqgan: Sequence generative adversarial nets with policy gradient. In: Proceedings of the AAAI conference on artificial intelligence. vol. 31; 2017.

42. Huang CZA, Cooijmans T, Roberts A, Courville AC, Eck D. Counterpoint by Convolution. In: International Society for Music Information Retrieval Conference; 2019.

43. Uria B, Murray I, Larochelle H. A deep and tractable density estimator. In: International Conference on Machine Learning. PMLR; 2014. p. 467 475.

44. Vasquez S, Lewis M. MelNet: A Generative Model for Audio in the Frequency Domain. arXiv preprint arXiv:190601083. 2019;.

45. Snoek J, Larochelle H, Adams RP. Practical bayesian optimization of machine learning algorithms. Advances in neural information processing systems. 2012;25.

46. Martinez-Cantin R, De Freitas N, Brochu E, Castellanos J, Doucet A. A Bayesian exploration-exploitation approach for optimal online sensing and planning with a visually guided mobile robot. Autonomous Robots. 2009;27:93–103.

47. Calandra R, Seyfarth A, Peters J, Deisenroth MP. Bayesian optimization for learning gaits under uncertainty: An experimental comparison on a dynamic bipedal walker. Annals of Mathematics and Artificial Intelligence. 2016;76:5 23.

48. Chen Y, Huang A, Wang Z, Antonoglou I, Schrittwieser J, Silver D, et al. Bayesian Optimization in AlphaGo. ArXiv. 2018:abs/1812.06855.

49. Forrester AI, Bressloff NW, Keane AJ. Optimization using surrogate models and partially converged computational fluid dynamics simulations. Proceedings of the Royal Society A: Mathematical, Physical and Engineering Sciences. 2006;462(2071):2177–2204.

50. Tamura R, Hukushima K. Bayesian optimization for computationally extensive probability distributions. Plos one. 2018;13(3):e0193785.

51. Czarnecki WM, Podlewska S, Bojarski AJ. Robust optimization of SVM hyperparameters in the classification of bioactive compounds. Journal of cheminformatics. 2015;7:1–15.

52. Wario F, Wild B, Couvillon MJ, Rojas R, Landgraf T. Automatic methods for long-term tracking and the detection and decoding of communication dances in honeybees. Frontiers in Ecology and Evolution. 2015;3:103.

53. Fleming JE, Pont Sanchis I, Lemmens O, Denison-Smith A, West TO, Denison T, et al. From dawn till dusk: Time-adaptive bayesian optimization for neurostimulation. PLOS Computational Biology. 2023;19(12):e1011674.

54. Nishio M, Nishizawa M, Sugiyama O, Kojima R, Yakami M, Kuroda T, et al. Computer-aided diagnosis of lung nodule using gradient tree boosting and Bayesian optimization. PloS one. 2018;13(4):e0195875.

55. Chong T, Shen IC, Sato I, Igarashi T. Interactive Optimization of Generative Image Modelling using Sequential Subspace Search and Content-based Guidance. Computer Graphics Forum. 2020;40. doi:10.1111/cgf.14188.

56. Koyama Y, Sato I, Sakamoto D, Igarashi T. Sequential line search for efficient visual design optimization by crowds. ACM Trans Graph. 2017;36(4). doi:10.1145/3072959.3073598.

57. Brochu E, Cora VM, De Freitas N. A tutorial on Bayesian optimization of expensive cost functions, with application to active user modeling and hierarchical reinforcement learning. arXiv preprint arXiv:10122599. 2010;.

58. Granley J, Fauvel T, Chalk M, Beyeler M. Human-in-the-loop optimization for deep stimulus encoding in visual prostheses. Advances in neural information processing systems. 2023;36:79376–79398.

59. Koide S, Okoso A. High-Dimensional Slider-Based Preferential Bayesian Optimization With Mixed Local and Global Acquisition Strategies. IEEE Access. 2024;12:90862–90872.

60. Hvarfner C, Hellsten EO, Nardi L. Vanilla Bayesian Optimization Performs Great in High Dimensions. In: Proceedings of the 41st International Conference on Machine Learning. vol. 235 of Proceedings of Machine Learning Research; 2024. p. 20793–20817.

61. Papenmeier L, Nardi L, Poloczek M. Increasing the scope as you learn: Adaptive Bayesian optimization in nested subspaces. Advances in Neural Information Processing Systems. 2022:35:11586–11601.

62. Eriksson D, Jankowiak M. High-dimensional Bayesian optimization with sparse axis-aligned subspaces. In: Uncertainty in Artificial Intelligence. PMLR; 2021. p. 493–503.

63. Duvenaud DK, Nickisch H, Rasmussen C. Additive gaussian processes. Advances in neural information processing systems. 2011;24.

64. Ziomek JK, Ammar HB. Are random decompositions all we need in high dimensional Bayesian optimisation? In: International Conference on Machine Learning. PMLR; 2023. p. 43347–43368.

65. Oh C, Gavves E, Welling M. BOCK: Bayesian optimization with cylindrical kernels. In: International Conference on Machine Learning. PMLR; 2018. p. 3868–3877.

66. Gulrajani I, Ahmed F, Arjovsky M, Dumoulin V, Courville AC. Improved training of wasserstein gans. Advances in neural information processing systems. 2017;30.

67. Raffel C. Learning-based methods for comparing sequences, with applications to audio-to-midi alignment and matching. Columbia University; 2016.

68. Harte C, Sandler M, Gasser M. Detecting harmonic change in musical audio. In: Proceedings of the 1st ACM workshop on Audio and music computing multimedia; 2006. p. 21–26.

69. Sobol' IM. On the distribution of points in a cube and the approximate evaluation of integrals. Zhurnal Vychislitel'noi Matematiki i Matematicheskoi Fiziki. 1967;7(4):784–802.

70. Shahriari B, Bouchard-Cote A, Freitas N. Unbounded Bayesian Optimization via Regularization. In: Proceedings of the 19th International Conference on Artificial Intelligence and Statistics. vol. 51 of Proceedings of Machine Learning Research. PMLR; 2016. p. 1168–1176.

71. Moriconi R, Deisenroth MP, Sesh Kumar K. High-dimensional Bayesian optimization using low-dimensional feature spaces. Machine Learning. 2020;109:1925–1943.

72. Ridnik T, Ben-Baruch E, Zamir N, Noy A, Friedman I, Protter M, et al. Asymmetric loss for multi-label classification. In: Proceedings of the IEEE/CVF international conference on computer vision; 2021. p. 82–91.

73. Wang Z, Bovik AC, Sheikh HR, Simoncelli EP. Image quality assessment: from error visibility to structural similarity. IEEE transactions on image processing. 2004;13(4):600–612.

74. Fu H, Li C, Liu X, Gao J, Celikyilmaz A, Carin L. Cyclical Annealing Schedule: A Simple Approach to Mitigating KL Vanishing. In: Proceedings of the 2019 Conference of the North American Chapter of the Association for Computational Linguistics: Human Language Technologies, Volume 1 (Long and Short Papers); 2019. p. 240–250.

75. Stewart GW. The efficient generation of random orthogonal matrices with an application to condition estimators. SIAM Journal on Numerical Analysis. 1980;17(3):403–409.

76. Feng Q, Guo C, Benitez-Quiroz F, Martinez AM. When do gans replicate? on the choice of dataset size. In: Proceedings of the IEEE/CVF International Conference on Computer Vision; 2021. p. 6701–6710.

77. Martinez-Cantin R. BayesOpt: A Bayesian Optimization Library for Nonlinear Optimization, Experimental Design and Bandits. Journal of Machine Learning Research. 2014;15:3735–3739.

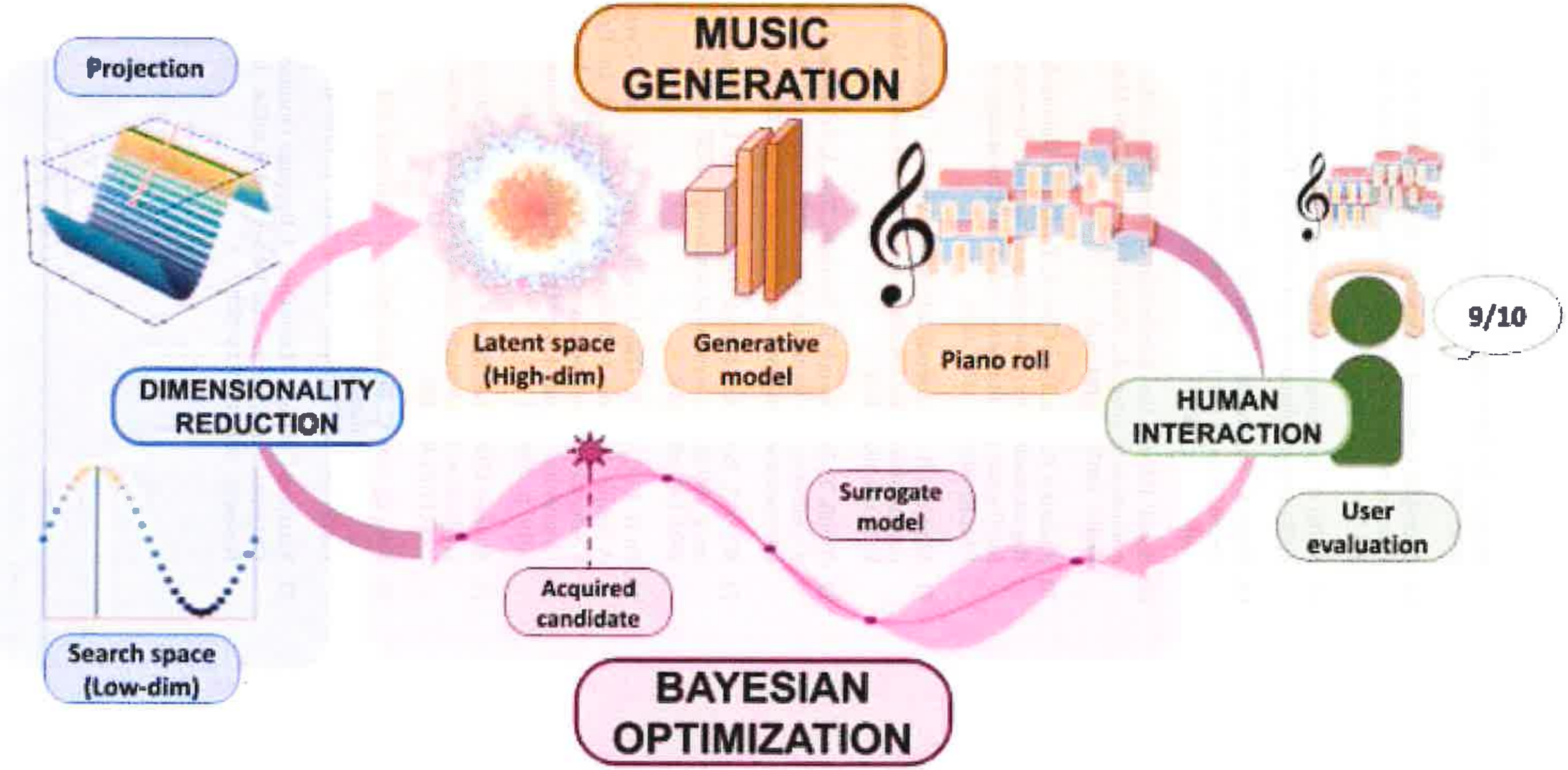
Projection
MUSIC GENERATION
Latent space (High-dim)
Generative model
Piano roll
9/10
DIMENSIONALITY REDUCTION
HUMAN INTERACTION
Surrogate model
User evaluation
Acquired candidate
Search space (Low-dim)
BAYESIAN OPTIMIZATION

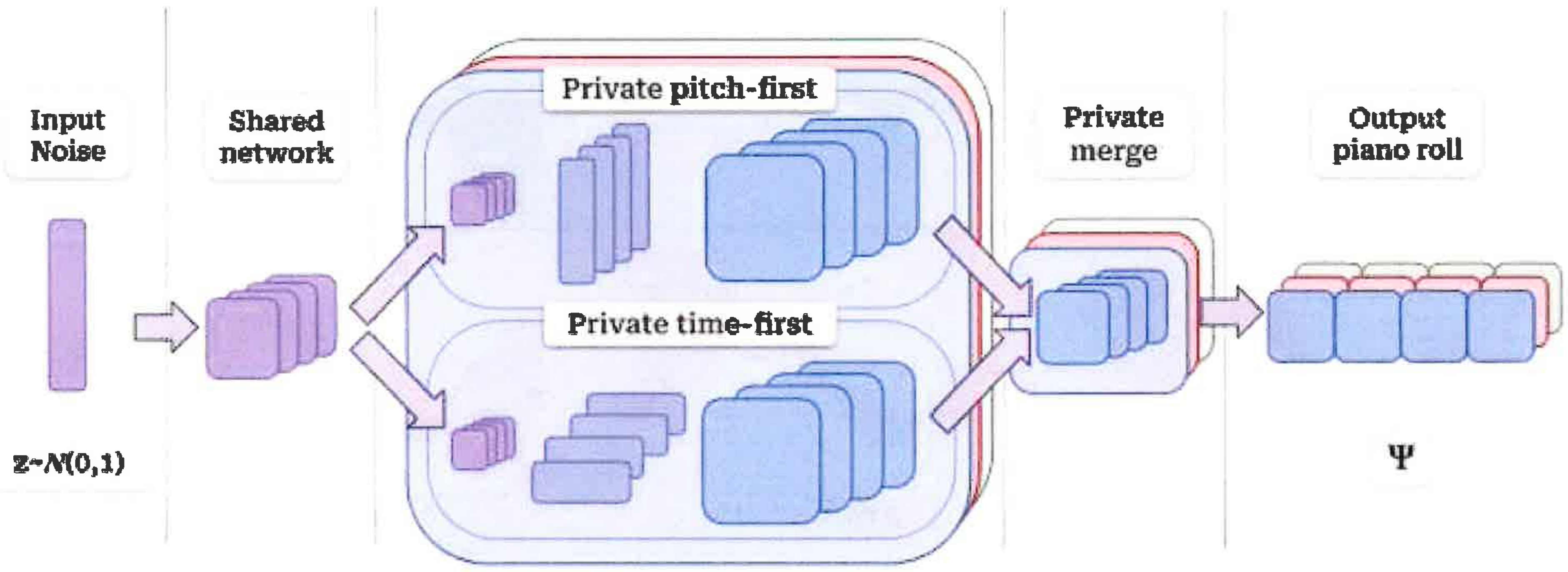
Input
Noise
Shared
network
Private pitch-first
Private time-first
Private merge
Output
piano roll
z~𝒩(0,1)
Ψ

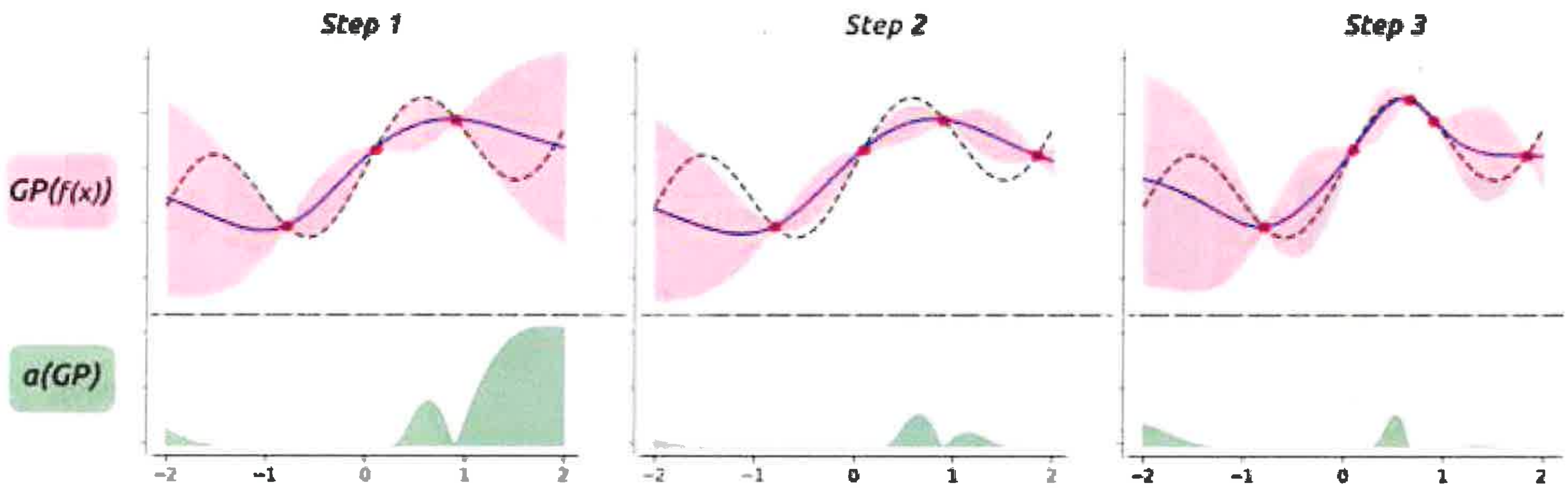
Step 1
Step 2
Step 3
GP(f(x))
a(GP)
−2
−1
0
1
2

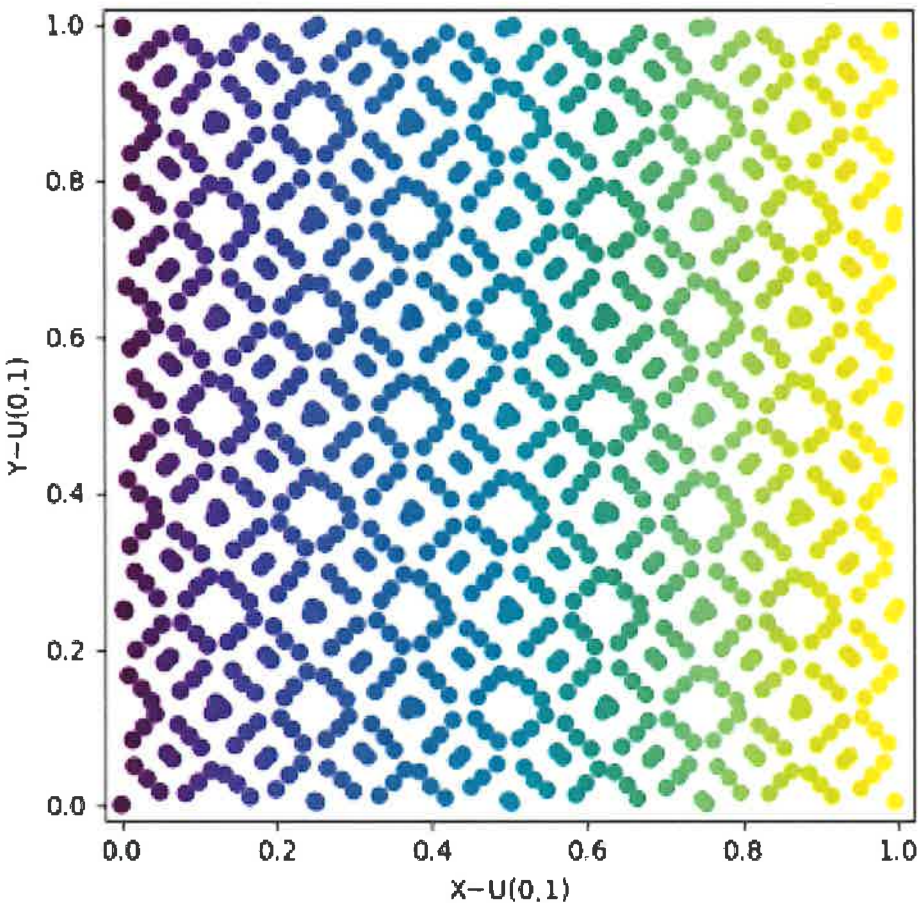
1.0
0.8
0.6
0.4
0.2
0.0
Y~U(0,1)
0.0
0.2
0.4
0.6
0.8
1.0
X~U(0,1)

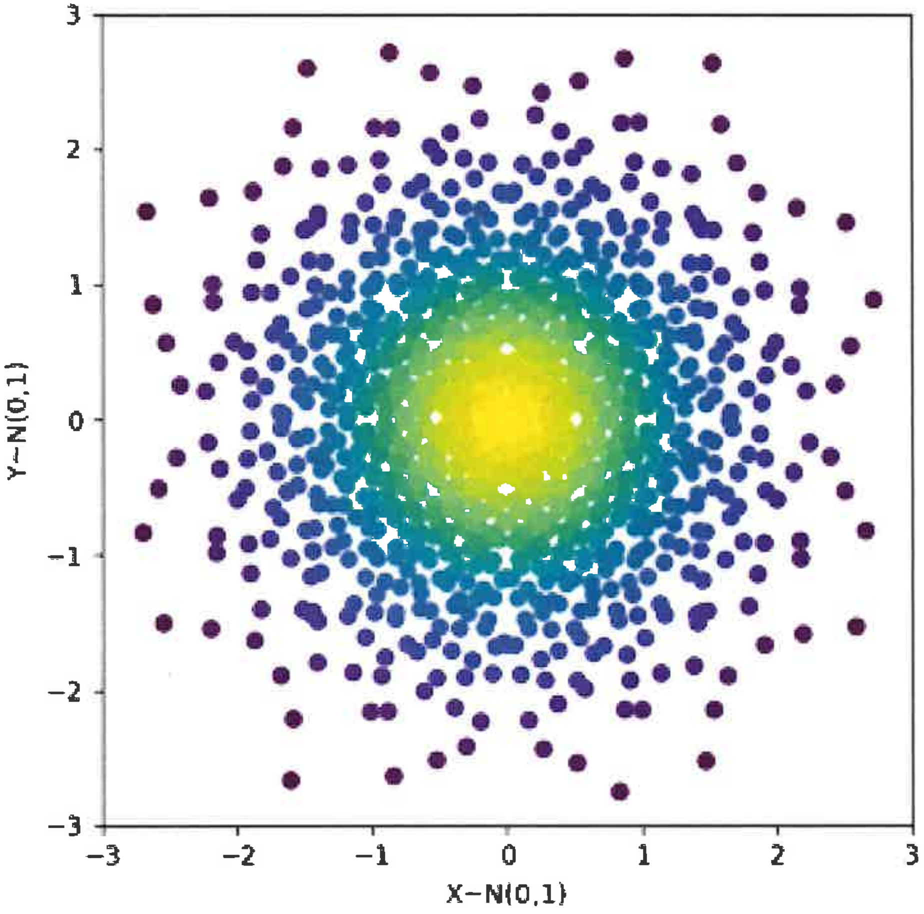
3
2
1
0
−1
−2
−3
Y~N(0,1)
−3
−2
−1
0
1
2
3
X~N(0,1)

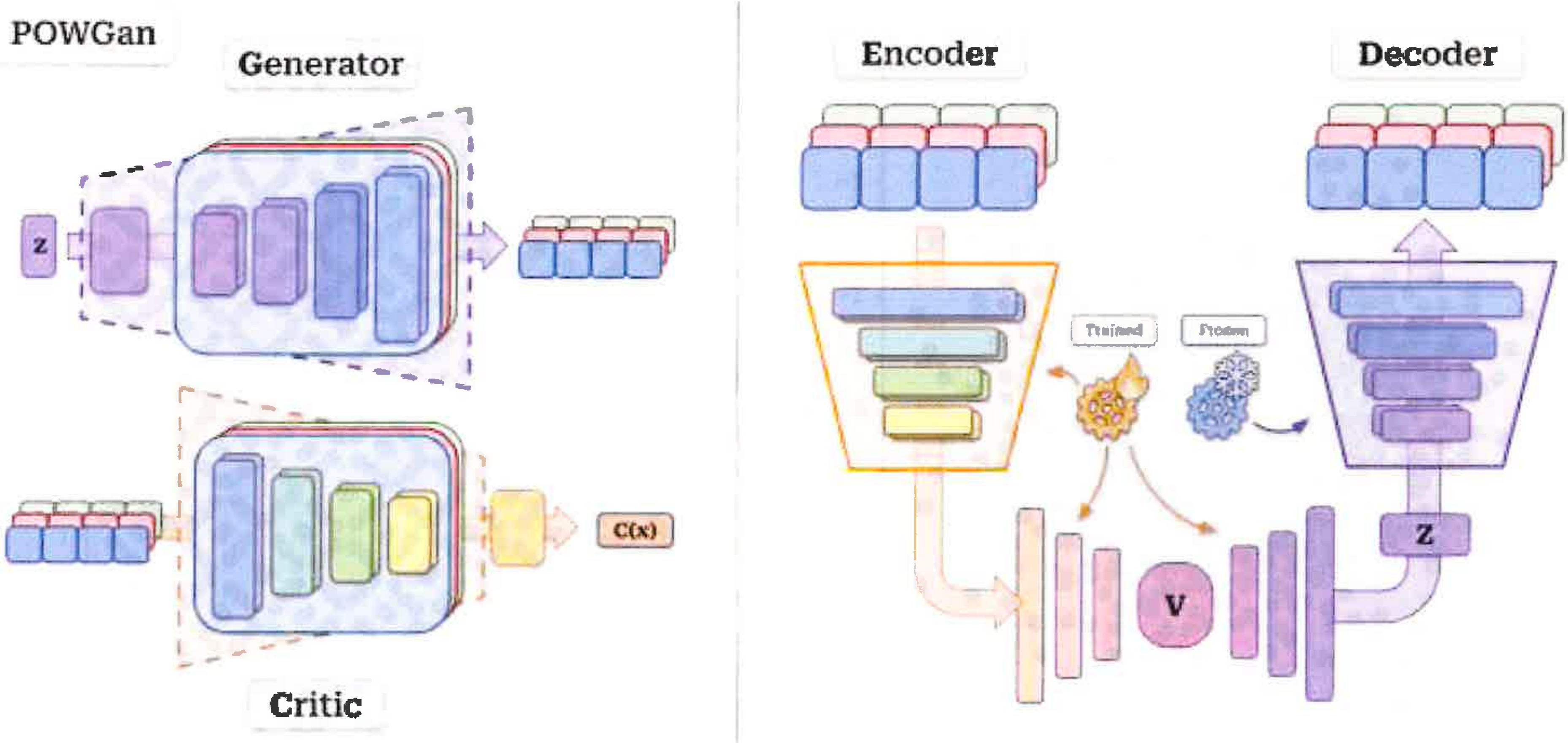
POWGan
Generator
z
C(x)
Critic
Encoder
Decoder
Trained
Frozen
V
Z

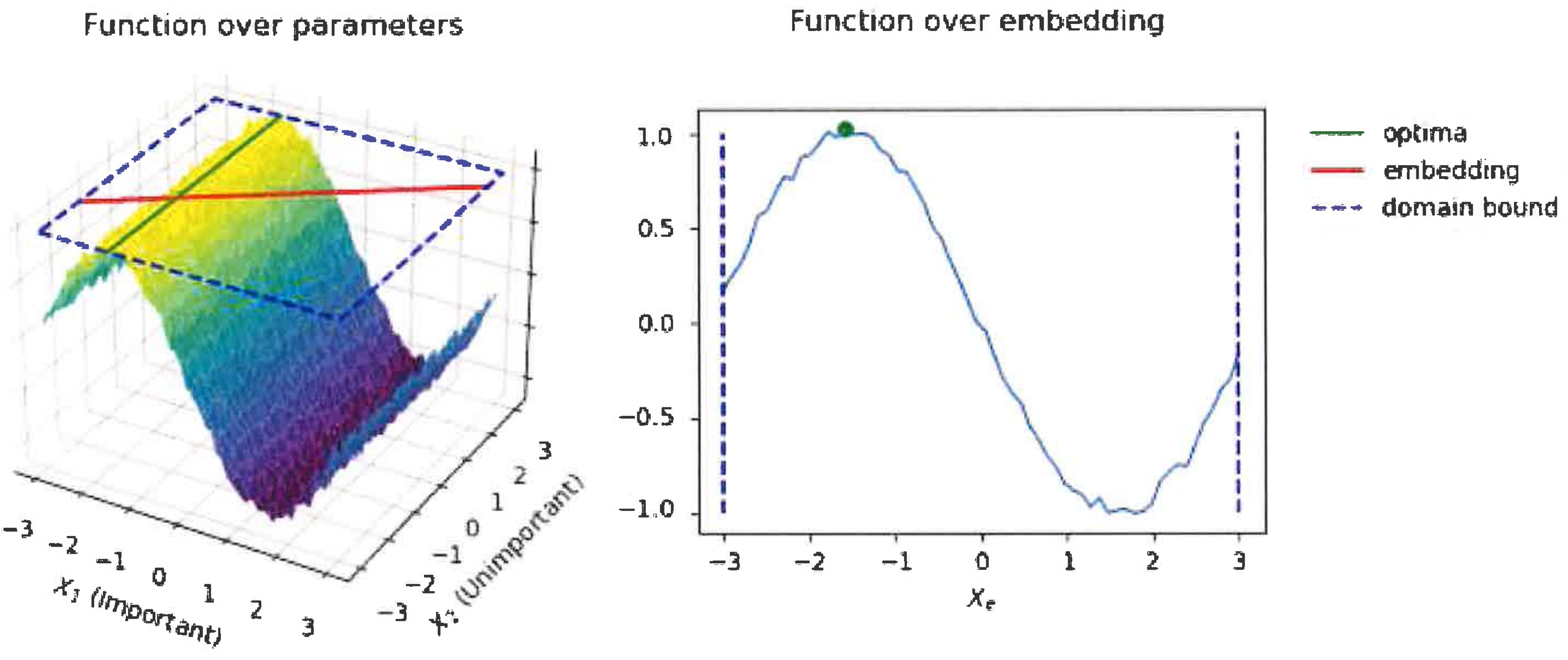
Function over parameters
X_1 (Important)
(Unimportant)
Function over embedding
X_e
optima
embedding
domain bound

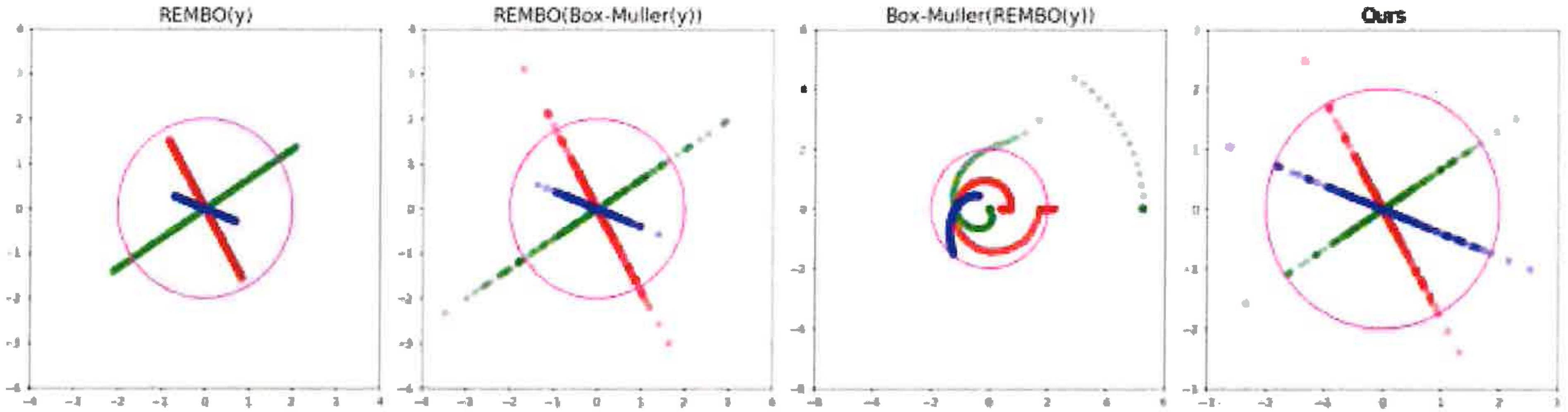
REMBO(y)
REMBO(Box-Muller(y))
Box-Muller(REMBO(y))
Ours

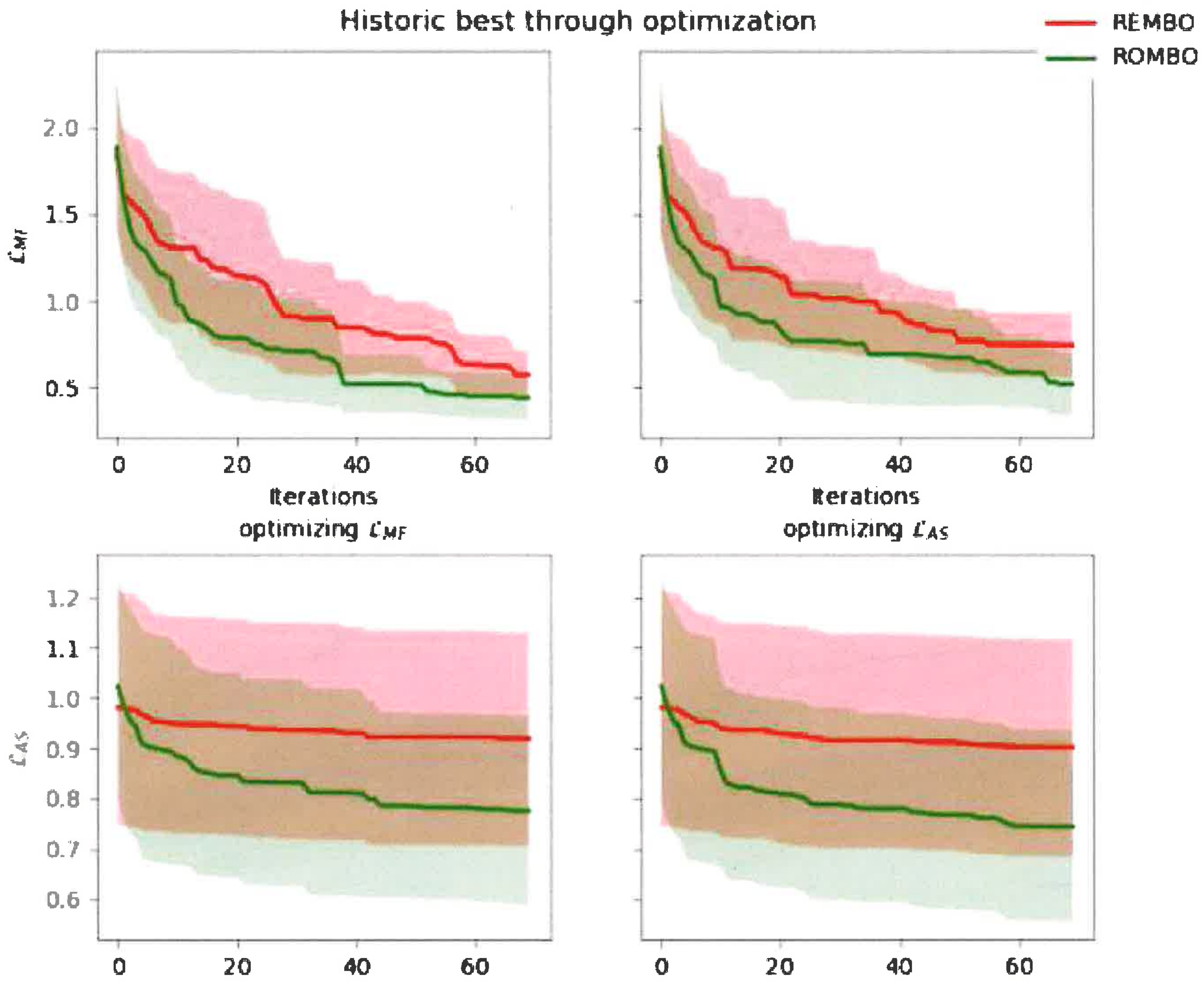

Historic best through optimization
REMBO
ROMBO
$\mathcal{L}_{MI}$
2.0
1.5
1.0
0.5
0
20
40
60
Iterations
optimizing $\mathcal{L}_{MF}$
optimizing $\mathcal{L}_{AS}$
$\mathcal{L}_{AS}$
1.2
1.1
1.0
0.9
0.8
0.7
0.6

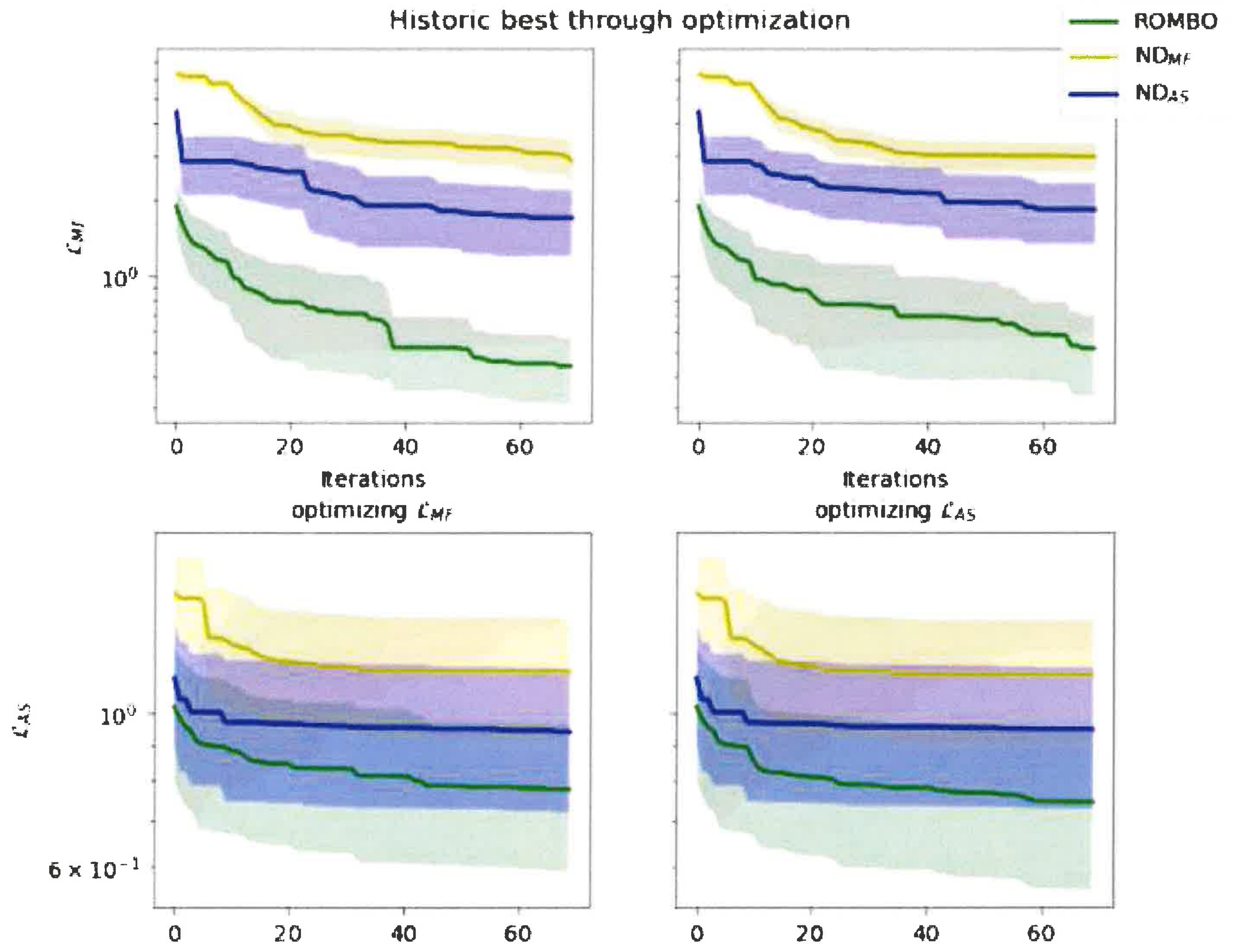

Historic best through optimization
ROMBO
ND_{MF}
ND_{AS}
$\mathcal{L}_{MF}$
10^0
0
20
40
60
Iterations
optimizing $\mathcal{L}_{MF}$
optimizing $\mathcal{L}_{AS}$
$\mathcal{L}_{AS}$
6×10^{-1}

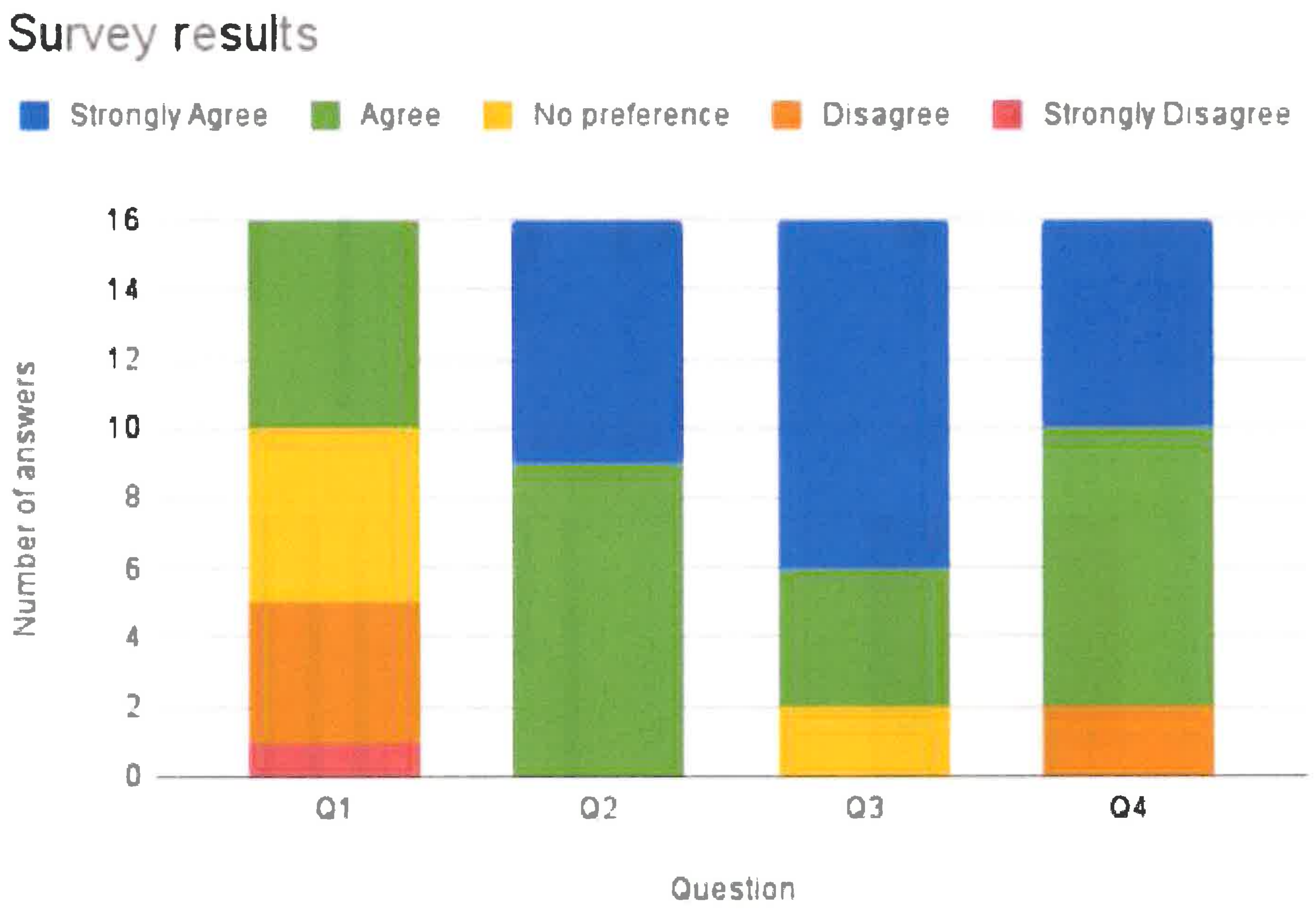
Survey results
Strongly Agree
Agree
No preference
Disagree
Strongly Disagree
Number of answers
16
14
12
10
8
6
4
2
0
Q1
Q2
Q3
Q4
Question

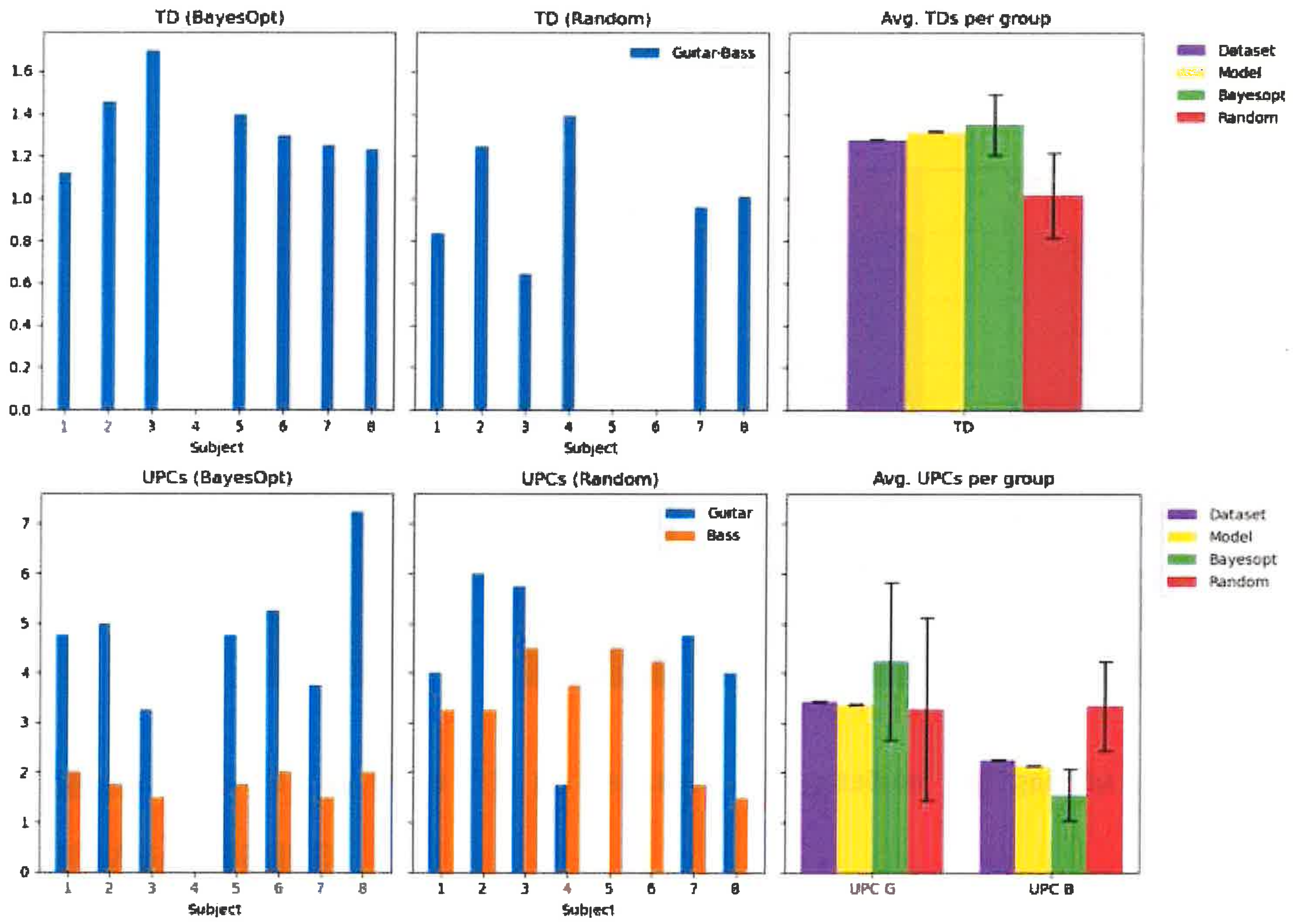
TD (BayesOpt)
TD (Random)
Avg. TDs per group
Guitar-Bass
Dataset
Model
Bayesopt
Random
Subject
TD
UPCs (BayesOpt)
UPCs (Random)
Avg. UPCs per group
Guitar
Bass
UPC G
UPC B

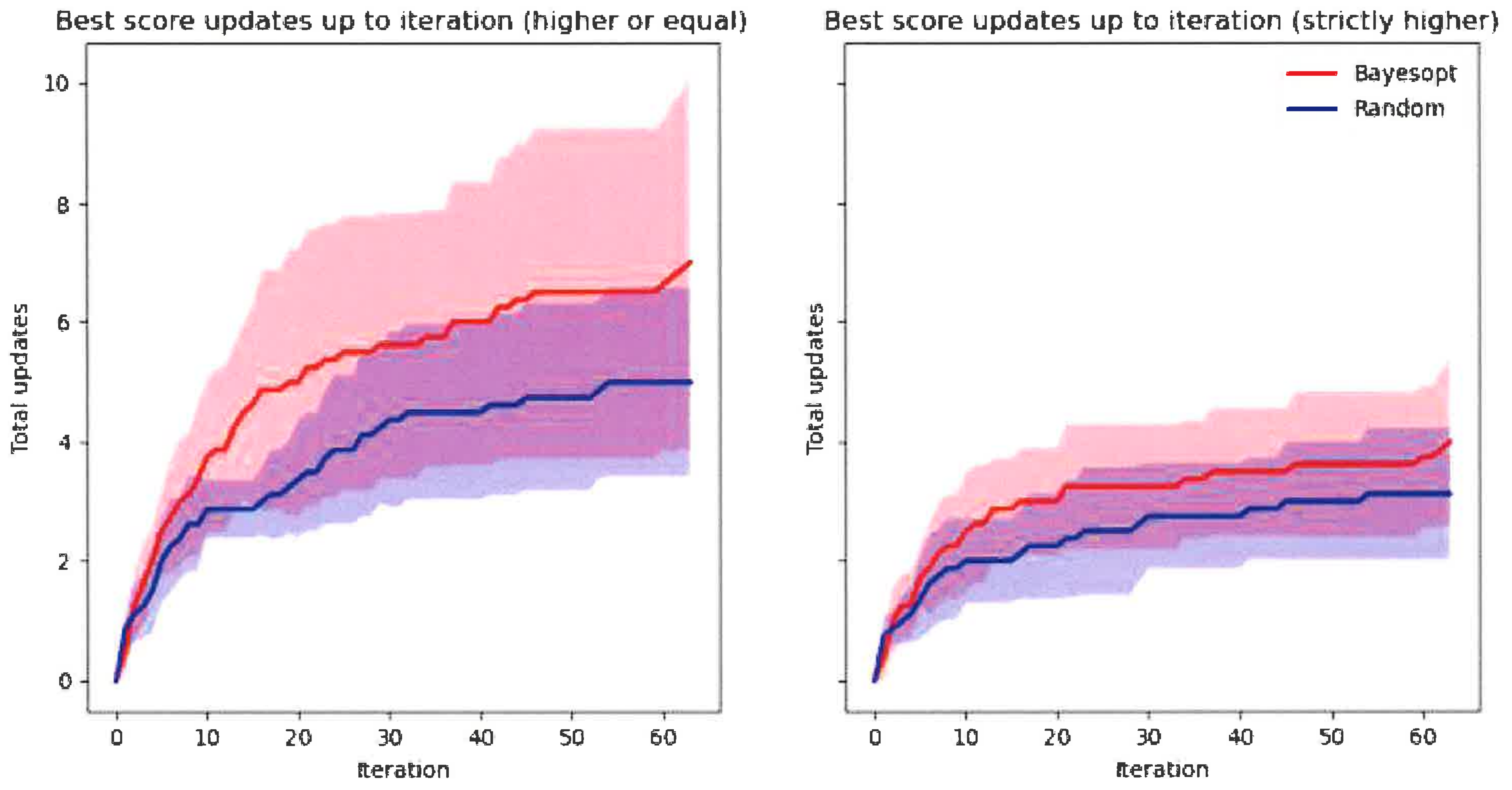

Best score updates up to iteration (higher or equal)
Total updates
10
8
6
4
2
0
0
10
20
30
40
50
60
Iteration
Best score updates up to iteration (strictly higher)
Bayesopt
Random
Total updates
0
10
20
30
40
50
60
Iteration
