# Supplementary material for: Random rotational embedding Bayesian optimization for human-in-the-loop personalized music generation
Source: PLoS One. 2025 Nov 21;20(11):e0335853. doi: 10.1371/journal.pone.0335853 (PMC12637961; doi:10.1371/journal.pone.0335853)
Supplement: Attachment — Submitted filename: Response to Reviewers.pdf [file pone.0335853.s002.pdf]

Subject: Response to reviewers of manuscript PONE-D-25-27090

Dear Dr. Tara Rajendran,

Please find attached the new version of our manuscript titled "Random rotational embedding Bayesian optimization for human-in-the-loop personalized music generation" to the PLOS One journal. We would like to thank the reviewers for their time and thoughtful comments. Those comments have helped us to improve our manuscript significantly. We have a detailed and individual response to the reviewers immediately after this letter. The changes have also been highlighted in red and blue in the manuscript.

More concretely:

- We have adjusted our data availability statement, and have released a GitHub pages site where several of our users' favorite generated songs can be found for the reviewers to listen to. Our code is also published in a public repository for reproducibility and transparency.

  https://github.com/mikceroese/ROMBO

- We have included a new simulated evaluation for random search, comparing our method and baselines against naïve approaches. We show how it performs poorly compared to our method, strengthening our claims.
- We have rewritten the abstract, introduction and conclusions focusing the scope on the algorithmic contribution of the paper. We have also extended the conclusion, addressing the limitations of our pilot user study and future work.
- We have revised our writing, correcting typos and following the reviewers' notes on improving readability.

We hope that the new version can be accepted for publication in PLOS One.

Sincerely,
Miguel Marcos

Reviewer Comments, Author Responses and Manuscript Changes

We would like to thank the reviewers for their time, praise, and comments. We have tried to address all the comments as best as possible. We have written in blue our response to each of the reviewers' comments.

Reviewer #1:

Comment 1: The abstract could benefit focusing on informative and concrete details. Likewise, it may help to list the paper's main contributions or findins clearly. For example, it could detail three main-claims (for example: (1) "We propose a [X%] more efficient embedding strategy"; (2) "We achieve [Y%] lower loss in simulation"; (3) "In a user study (n=16), preference scores improved by [Z points]"). Similarly, conclusion might benefit if it explicitly acknowledges limitations (sample size) and outlines next steps to end on a positive-holding suggestion for future work.

Response: Thank you for your suggestions. We have revisited the abstract, highlighting the main points of the paper and providing the results snippet as you commented. As for the conclusion, we have added discussion about the limitations and future lines of work.

Comment 2: Even as full of content, some sentences in the draft might seem overly long and dense. For example, the abstract's one-sentence description of the listener task tries to cover background, method, and goal all at once. Some frameworks recommend aiming for roughly 12-15 words per sentence in academic writing. Define specialized terms. The flow of the introduction can be improved by breaking up large paragraphs and using clear headings.

Response: Thank you for your advice. We have reviewed the paper in search of overly long sentences and rewritten them for clarity. We have also restructured the introduction to improve the reading flow.

Comment 3: The Methods section may benefit from intuitive explanations of technical formulas, kernels and prior models. When introducing kernel, the text shows unintuitive equation. It might help to say in plain language what it does. Likewise, the paper currently cites classical strategies and additive models, but could mention several emerging approaches in high-dimensionality. Sparsity priors might model automatically ignoring irrelevant inputs and prior sparse axis-subspaces alineation could induce prior-shrinkage upon each kernel lengthscale.

Response: We have rewritten the GP section to make it more accessible, while keeping it formal. Thank you for the pointers to the related work. As far as we can tell, the only works that we know about sparsity priors are those of Eriksson et al., which we already cited in the original manuscript, and Papenmeier et al. which is still an arXiv preprint. However, we cite the new PhD thesis from Papenmeier published this June. Note that sparsity priors could actually be complementary to our rotational embedding, making it a potential

line of future work. We have included this in our conclusions.

Comment 4: Regarding evaluation baselines, it could be relevant to consider a simple control/null method for comparison. If even a naïve search outperforms no embedding, it underscores the need for advanced methods.

Response: We have included a random baseline for comparison in Figure 8, and updated the discussion in this section. Initially, we also tried vanilla BO in 256 dimensions, but it never improved over the initial points, having really bad performance, even worse than random. We have not included the results for the sake of clarity on the plots, but mentioned them in the text.

Comment 5: The paper could connect more explicitly how the latest Transformer- and Diffusion-based music generation models achieve high realism from text prompts but usually lack personalization loops. [..] In the discussion or future work, it would be valuable to mention diffusion models explicitly. Inviting integration of the pipeline with diffusion-based music generators would suggest that the proposed optimization could plug into cutting-edge audio pipelines.

Response: Note that our optimization pipeline is **agnostic of the generative model used**. Bayesian optimization has been called a method for black-box optimization, meaning that it does not require knowledge about the function to be optimized. Our rotational embedding works with any Gaussian latent space, which is the standard practice in all generative models, independent of whether they are based on transformers, GANs, diffusion, etc. We have explained more clearly the agnosticism of our method in the 'High-dimensional Bayesian optimization' subsection. Nonetheless, we have also extended the future work discussion in the conclusion to include other generative models.

Comment 6: Another growth idea is evaluating creativity and diversity. Mode collapse (generating many similar samples) is a known risk in generative models. The authors could guard against it by measuring diversity in the top-ranked songs, computing an entropy or inter-sample distance metric over the high-rated outputs.

Response: A *feature* of our approach is that we have no control on the diversity of the generated results, only on the diversity of the generated samples in the latent space. Mode collapse occurs when the model generates similar outputs with different internal samples. As discussed before, one of the advantages of our method is that it uses a black-box approach, meaning that we can use any model, but at the same time, we cannot interfere with the model. Our Bayesian optimization method does guarantee that the internal samples are actually diverse enough as the expected improvement automatically trade off exploration and exploitation. For the experiments, we use a Wasserstein GAN which is a generalization of a GAN specifically designed to minimize mode collapse on the output.

Comment 7: Finally, the human study could be enriched with qualitative

feedback if available. In addition to numeric ratings, gathering open-ended comments or interview data can reveal how users perceive and engage with the generative process. Adding this perspective helps align with good practices and strengthens the human-centric claims of the paper.

Response: We purposely design the experiment in such a way that all the data can be studied in aggregated form for privacy concerns, as requested by our ethics committee. In this work, the human study is to evaluate the performance of our algorithm contribution and the viability of the personalization process. User engagement is beyond the scope of the paper. Nonetheless, we have considered this line as future work in our conclusions.

Comment 8: To further align with open science norms, the authors should include - if feasible - song files, evaluation scripts, and survey results in a public repository. [..] Adjustments like clear data-code sharing, compute details, seeds, and demographics could enhance the manuscript's rigor and reproducibility and show attention to openness, allowing the community to verify and build on the work, aligning with current best practices in AI research.

Response: Thank you for your recommendations. We have updated our availability statement and **released our code and song files in a public repository**. Some of the items in this list (demographics and survey results) were already included in the first version of the manuscript. For privacy reasons, we cannot disclose individual surveys. Hardware and compute times are reported in the *Pipeline Evaluation* section. We revised the text of these sections, including the mean age of the participants, and updated Figure 10, which features the survey results, to increase visibility.

Reviewer #2:

Comment 1: Evaluation for the algorithm's performance, and a subsequent "real-world" experiment for satisfaction levels against controls, argue quite convincingly for the effectiveness of this novel application. It is basically well written, although please revise for some inconsistent and inaccurate use of singular/plural cases in the written language.

Response: Thank you for your kind review. We are happy to read that our algorithm evaluation convinced you of its worth. We have revisited our writing as you suggested to improve the readability of our paper. We hope to see it accepted after this revision.

Reviewer #3:

Comment 1: The sample size is a major concern. With only 16 participants, the statistical power is very limited even when using Bayesian approaches. In a simple within-subject design, a minimum of 20–30 participants with 30–50 trials per participant is generally expected, especially when relying on limited prior information. As the authors describe the study as a novel setup involving multiple new factors, the rationale for the small sample size is unconvincing.

The manuscript mentions 22 samples for the Sobol analysis and 52 samples for optimization.

Response: Thank you for your comment. First, please note that the main contribution of the paper is a new algorithm, with its corresponding mathematical proof and software implementation. The human study is a way to validate the algorithm component, not the main contribution. Second, in Bayesian optimization literature, our study is much larger than similar studies. The only other work that did human-in-the-loop optimization for music generation [29] had just 2 participants. In Kim et al. [15], published in Plos ONE as well, they had 9 participants. In Lorenz et al. [57], only 12 people participated in the study. Third, we are not doing hypothesis testing. Bayesian optimization algorithms, like many probabilistic optimization and reinforcement learning algorithms, are always compared based on averaged performance (see ALL the citations in the Bayesian optimization related work section). Fourth, it should be noted that, as you mention, for any participant, we generated 64 (22+42, not 52) different songs. Thus, the number of data points is actually 16x64=1024, which is just above the average of the ranges that you suggested (25 participants x 40 trials = 1000 data points). We have rewritten the manuscript, especially the abstract and introduction, to highlight and stress the main contribution and orientation of the paper.

Comment 2: The paper does not include formulated hypotheses or assumptions, either before or following the results section. This absence makes it difficult to evaluate the coherence and direction of the research. The conclusion appears vague and underdeveloped. While the authors suggest that their model "works", there is little in-depth explanation as to why this is the case or how the research and control groups differ meaningfully. More interpretation and reflection on the findings are needed. The role and use of the control group are somewhat confusing. Please elaborate on its function and how the results differ between the experimental and control groups.

Response: As stated before, this is an algorithmic paper that typically does not include a hypothesis, as the purpose is always to define a new method and validate if it "works". However, in order to make the manuscript more accessible to a larger audience, we have included a paragraph explicitly stating the hypothesis in the introduction section. Our hypothesis is twofold. First, Bayesian optimization using rotational embeddings outperforms the state-of-the-art when faced with a high-dimensional Gaussian input space, such as the sampling space in generative models. Second, Bayesian optimization with our rotation embedding can be used to personalize preferences in generative models.

As we explain in our **Experimental design** section, the control group has no optimization pipeline running. No information about whether they like the pieces or not reaches the model, so the produced songs are random. We can consider the control group as having a *placebo* algorithm that actually *does nothing* in terms of personalization. The results of the experiment, reported in Table 6, Figures 10 and 11, and the text in their section, show that users in the research group lean towards more likable pieces. To further increase visibility,

we have rewritten our abstract, featuring the exact numerical improvements, and extended our comments on them in the Conclusion as well. The first part of the hypothesis is **proven mathematically** and validated with simulated data, as the hypothesis is about the numerical comparison of algorithms.

Note that what we are validating is the personalization/optimization part and the embedding. The generative model that we use was already validated in the original work [26].

Comment 4: The manuscript requires substantial language editing. There are grammatical issues, unclear phrasing, and inconsistent use of tenses, sometimes even within the same sentence. For example: "After collecting the volunteer's consent, they are told the following pieces of information." Such inconsistencies hinder readability and should be addressed throughout the paper.

Response: Thank you for pointing out the grammatical mistakes. We have rewritten the manuscript, removing any inconsistencies.

Comment 5: Minor writing comments:

Response: As discussed already to reviewer 1, we have written a new abstract that features these improvements, highlighting the main conclusions and explicitly stating the number of participants and results from the user study.

The introduction has also been rewritten to further clarify the type of music generation used and the purpose of the paper. Actually, this work is a preliminary step on a much larger project on audio therapy, so our intent could not be further from the commercial applications mentioned by the reviewer.

We have also followed your recommendations about writing style for the method and conclusion sections. We remade Figure 10 as well to unify the used software.

Note that, for algorithmic papers, the standard convention is the presentation of the **contributions** at the end of the introduction and the **lack of a discussion section**, which, according to the author guidelines of the journal, is completely optional. See for example [45,46] published both in PLOS One as well.

Reviewer #4:

Comment 1: As a musician with a keen (albeit new since 2022) interest in artificial intelligence, I found this manuscript fascinating despite not always understanding all of the mathematics. I would have liked to see the data and analysis on Github prior to acceptance, but I understand your rationale for not wanting to share everything right away.

Response: Thank you for your kind review. Regarding the data release, we have decided to update our data availability statement and released a GitHub pages site where you can now listen to some samples. We include both random generations of the model and our Bayesopt group users' best graded generations.

Comment 2: As interesting as this work was, and as much as I learned just by reading it, I do wonder what the purpose is of "artificially generating music." In general, why try to get a machine (or an algorithm or a gpt) to make music? Why isn't human generation of music enough?

Response: We are happy to be of interest, and aware of the potential for debate of music generation. First of all, please bear in mind that our contribution is mainly algorithmic and, more importantly, model-agnostic. Music generation is a high-dimensional problem, subjective, and in need of efficiency. These traits combined make it quite a unique problem, but not our sole purpose. Any other task that shares them - and involves Gaussian latent spaces - can benefit from our findings. We are "computer scientists with a keen interest in music". Besides, as discussed with reviewer 3, the use of music specifically is a preliminary step in a larger project about audio therapy. However, the results are still too preliminary to present a viable connection.

Having said that, we do cite some other use cases in our **Introduction** section, like helping novice musicians. Our pipeline can be added, for example, to music editing software. Our study features a model that generates music from scratch, but since our algorithm is model-agnostic, it can be replaced with one that helps edit or complete human-generated music. We have made mention of this particular example in our Conclusions to help clear your and any other reader's doubts.

Comment 3: On page 5, I do ask a specific question related to musical complexity: If human music taste is subjective (and it definitely is), why should complexity matter?

Response: We acknowledge the wording may lead to misunderstanding. The term "complexity" here is used in its mathematical sense, meaning it adds too many dimensions to the problem. We have changed the phrasing in the manuscript to avoid confusion. Intuitively, if a person is to pay attention to five instruments at once, it may be difficult for them to actually listen closely to all of them. We decided that, from a user perspective, it would be easier to rate a three-track composition than a five-track one.

Comment 4: I am surprised that you do not mention the "uncanny valley" phenomenon as it applies to artificially generated sound, given that on page 16, you state that most participants do not explicitly like the generated songs. Maybe a follow-up study could be to find out WHY they don't like the songs?

Response: For the uncanny valley phenomenon, we are aware of it. However, it is usually tied to (not quite) human features, so in the audio domain it would apply to voice, which we do not generate. Due to limited computational resources, our model does not generate fully realistic audio waves. Instead, it generates MIDI files, which can actually be too artificial for many people. We believe that this would be the main source of people disliking the music. We have clarified that in the text. We invite you to listen to our released tracks on our GitHub page and judge for yourself how far we are from the uncanny valley.

The beauty of our optimization method is that, by presenting alternatives,

people can find their preferred song even if they do not know why. However, we share your opinion on how a follow-up study researching why songs are liked or not would be of interest.
